# Host and viral determinants of airborne transmission of SARS-CoV-2 in the Syrian hamster

**DOI:** 10.1101/2022.08.15.504010

**Published:** 2022-08-15

**Authors:** Julia R. Port, Dylan H. Morris, Jade C. Riopelle, Claude Kwe Yinda, Victoria A. Avanzato, Myndi G. Holbrook, Trenton Bushmaker, Jonathan E. Schulz, Taylor A. Saturday, Kent Barbian, Colin A. Russell, Rose Perry-Gottschalk, Carl I. Shaia, Craig Martens, James O. Lloyd-Smith, Robert J. Fischer, Vincent J. Munster

**Affiliations:** 1. Laboratory of Virology, Division of Intramural Research, National Institute of Allergy and Infectious Diseases, National Institutes of Health, Hamilton, MT, USA; 2. Department of Ecology and Evolutionary Biology, University of California, Los Angeles, CA, USA; 3. Rocky Mountain Research and Technologies Branch, Division of Intramural Research, National Institute of Allergy and Infectious Diseases, National Institutes of Health, Hamilton, MT, USA; 4. Department of Medical Microbiology | Amsterdam University Medical Center, University of Amsterdam; 5. Rocky Mountain Visual and Medical Arts Unit, Research Technologies Branch, Division of Intramural Research, National Institute of Allergy and Infectious Diseases, National Institutes of Health, Hamilton, MT, USA; 6. Rocky Mountain Veterinary Branch, Division of Intramural Research, National Institute of Allergy and Infectious Diseases, National Institutes of Health, Hamilton, MT, USA

## Abstract

Airborne transmission is one of the major routes contributing to the spread of SARS-CoV-2. Successful aerosol transmission occurs when people release respiratory particles carrying infectious virus in the fine aerosol size range. It remains poorly understood how infection influences the physiological host factors that are integral to this process. Here we assessed the changes in breathing, exhaled droplets, and released virus early after infection with the Alpha and Delta variants in the Syrian hamster. Infection with the two variants led to only nuanced differences in viral tissue titers, disease severity, or shedding magnitude. Both variants led to a short window of detectable virus in the air between 24 h and 48 h, which was poorly reflected by upper respiratory shedding measured in oropharyngeal swabs. The loss of viable air samples coincided with changes in airway constriction as measured by whole body plethysmography, and a decrease of fine aerosols produced in the 1–10 μm aerodynamic diameter range. We found that male sex was associated with greater viral replication in the upper respiratory tract and virus shedding in the air. This coincided with an exhaled particle profile shifted towards smaller droplets, independent of variant. Transmission efficiency of Alpha and Delta did not differ on average but exhibited clear variation among donor individuals, including a superspreading event. Transmission leading to substantial dual infections only occurred when both viruses were shed by the same donor and exposure was prolonged. These findings provide direct experimental evidence that quantitative and qualitative assessment of exhaled aerosols may be critical for understanding the limitations and determinants of efficient airborne transmission, thus allowing us to control the pandemic with non-pharmaceutical interventions.

## Introduction

Transmission by aerosolized virus particles has been a major contributor to the spread of SARS-CoV-2 [1, 2] [3–6]. Although highly efficient in preventing severe disease, vaccines do not significantly reduce transmission of variants of concern (VOCs) [7]. The initial Wuhan variant of SARS-CoV-2 (Lineage A) was overtaken by a lineage with the D614G mutation, then replaced by B1, which was replaced by Alpha [8], then, again replaced by Delta. Delta is suggested to derive its transmission advantage from increased furin cleavage and binding of spike to human ACE2 [9, 10].Transmission occurs when people release respiratory droplets carrying virus during (e.g.) speaking, singling, breathing, sneezing, or coughing. Droplet size and half-life in the air are not uniform [11, 12] and depend on speech and breathing patterns [13], COVID-19 severity, and physiological parameters such as age [14, 15]. As with influenza [16], SARS-CoV-2 RNA was detectable mostly in fine aerosols in humans, as opposed to coarse [14]. It is not clear how exhaled droplet size, breathing patterns and even the quantity of exhaled infectious virus itself fundamentally contribute to the airborne transmission efficiency *in vivo*. It remains poorly understood how COVID-19 directly influences additional physiological factors which may contribute to fine aerosol production. There is reportedly large heterogeneity in the transmission potential of individuals. Superspreading events have been reported numerous times throughout the pandemic and are suggested to be a major driver [17, 18]. They are thought to arise from a combination of biological, social, and chance factors. While human epidemiology and modeling studies have highlighted various factors which may contribute to SARS-CoV-2 transmission heterogeneity, including viral load [19], much of the observed variance remains poorly understood.

While inferences can be made from human studies, these factors are currently best studied in small animal models like the Syrian hamster, which allow for stringent and controlled experimental comparisons. The Syrian hamster model has been widely used to study SARS-CoV-2 transmission [20]; it recapitulates human contact, fomite and importantly, airborne short distance and fine aerosol transmission [21–24]. In this model the window of infectious virus shedding which can facilitate transmission has been shown to be limited. Highest efficiency of short-distance airborne transmission was observed before onset of weight loss and acute lung pathology, peaking at one day post inoculation and correlating to the highest virus loads in the upper respiratory tract of donor animals [25]. Data on lung function loss in the Syrian hamster model after SARS-CoV-2 infection is available [26, 27], and virus has been demonstrated in exhaled droplets [28]. Yet, a systematic study that addresses how airborne transmission potential depends on these features, along with recognized influences of sex and VOC, has not been performed. An experimental model to study these contributing factors of transmission heterogeneity would allow us to address how they come together to shape transmission outcomes.

Here, we correlated infectious virus released in the air with physiological parameters during SARS-CoV-2 infection for Alpha (B.1.1.7) and Delta (B.1.617.2). We introduce a mathematical model delineating the relationship between exhaled infectious virus and virus detected in the upper respiratory tract during infection with SARS-CoV-2, and longitudinally detail the changes in lung function, respiratory capacity, and exhaled particle profiles. Finally, we assess the airborne transmission competitiveness and heterogeneity *in vivo* of SARS-CoV-2 variants Alpha and Delta.

## Results

### SARS-CoV-2 Alpha and Delta spike display comparable entry phenotypes with human and hamster ACE2

**Figure 1.**
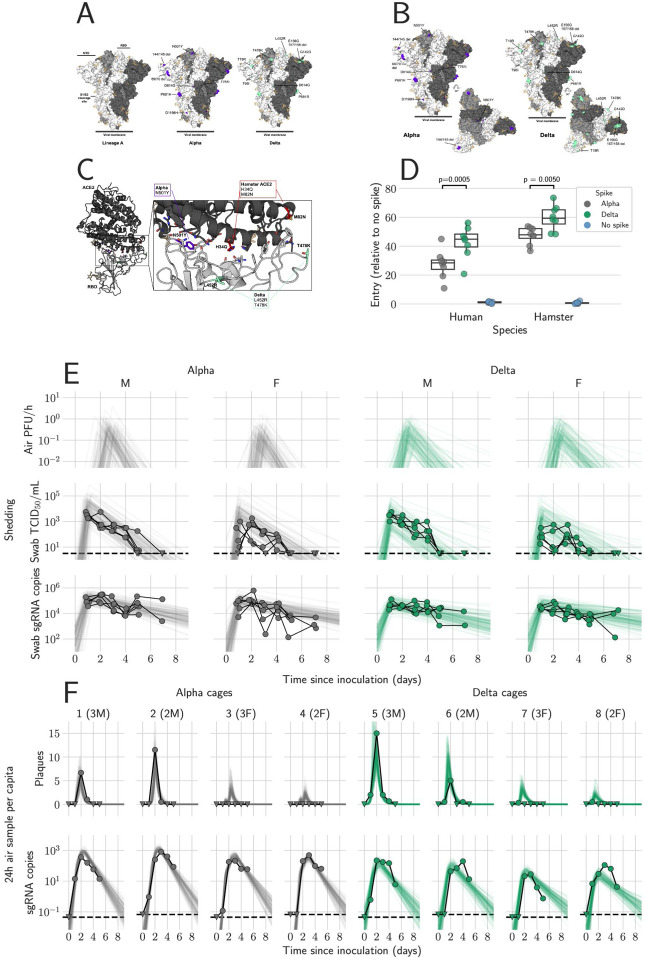
Alpha and Delta variant shedding profiles in oral swabs and air samples. Syrian hamsters were inoculated with 10^3^ TCID_50_ via the intranasal route with Alpha or Delta. **A/B.** Mutations observed in the SARS-CoV-2 Alpha and Delta VOCs are highlighted on the structure of SARS-CoV-2 spike (PDB 6ZGE, [53] A). The spike trimer is depicted by surface representation with each protomer colored a different shade of gray. The residues at the positions of the spike protein mutations observed in the Alpha and Delta SARS-CoV-2 VOCs are colored purple (Alpha) and teal green (Delta) and annotated. N-linked glycans are shown as light, orange-colored sticks. **C.** The structure of the Alpha VOC RBD and human ACE2 complex (PDB 7EKF [29]) is depicted with cartoon representation. ACE2 is colored dark gray and the RBD is colored light gray. N-linked glycans are shown as light, orange-colored sticks. A box reveals a close-up view of the RBD-ACE2 binding interface. Side chains of the residues participating in the interaction, as identified and described by Lan, et al. [30] are shown as sticks. The residues within the RBD that are mutated in the Alpha and Delta VOCs are colored purple (Alpha, N501Y) and teal green (Delta, L452R and T478K). Though they do not participate directly in the ACE2 interface, the sidechains of residues L452 and T478 are also shown. The residues that differ between human and hamster ACE2 within the interface are colored red. **D.** BHK cells expressing either human ACE2 or hamster ACE2 were infected with pseudotyped VSV reporter particles with the spike proteins of Alpha or Delta. Relative entry to no spike control is depicted. Boxplot depicting median, 95% CI and individuals, N = 8, ordinary two-way ANOVA, followed by Šídák’s multiple comparisons test. **E.** Comparison of swab viral load and virus shedding into the air. Inferred profile of air shedding in PFU/h compared to sgRNA levels and infectious virus titers (TCID_50_/mL) in oropharyngeal swabs collected 1, 2, 3, 4, 5 and 7 DPI. Semitransparent lines are 100 random draws from the inferred posterior distribution of hamster within-host kinetics for each of the metrics. Joined points are individual measured timeseries for experimentally infected hamsters; each set of joined points is one individual. Measurements and inferences shown grouped by variant and animal sex. Measurement points are randomly jittered slightly along the × (time) axis to avoid overplotting. **F**. Viral sgRNA and infectious virus (PFU) recovered from cage air sample filters over a 24 h period starting at 0, 1, 2, 3, 4, and 5 DPI. Points are measured values, normalized by the number of hamsters in the cage (2 or 3) to give per-capita values. Downward-pointing arrows represent virus below the limit of detection (0 observed plaques or estimated copy number corresponding to Ct ≥ 40). Semitransparent lines are posterior predictions for the sample that would have been collected if sampling started at that timepoint; these reflect the inferred underlying concentrations of sgRNA and infectious virus in the cage air at each timepoint and are calculated from the inferred infection kinetics for each of the hamsters housed within the cage. 100 random posterior draws shown for each cage. Cages housed 2 or 3 hamsters; all hamsters within a cage were of the same sex and infected with the same variant. Column titles show cage number and variant, with number of and sex of individuals in parentheses. Dotted lines = limit of detection. Grey = Alpha, teal = Delta, beige = anesthesia control, p-values are indicated where significant. Abbreviations: g, genomic; sg, subgenomic; TCID, Tissue Culture Infectious Dose; PFU, plaque forming unit; F, female; M, male.

Alpha and Delta possess several mutations in the spike protein compared to older strains, including changes in the receptor binding domain (RBD), N-terminal domain (NTD), and the S1/S2 cleavage site (Figure 1A,B) [29]. Of the ACE2 residues that directly participate in SARS-CoV-2 RBD binding, the histidine and methionine residues at positions 34 and 82 in human ACE2 are replaced by glutamine and asparagine in hamster ACE2 [2] [30]. (Figure 1C, red). These residues are not located in the immediate vicinity of either the Alpha or Delta VOCs mutations. It is plausible that both VOCs will similarly display increased entry using hamster ACE2. Both the Alpha N501Y mutation (Figure 1C, purple), and the two RBD mutations in Delta, L452R and T478K, are located away from the two substitutions in hamster ACE2 (Figure 1C, teal green). However, indirect effects resulting from these substitutions not reflected in the structural modelling cannot be ruled out. Therefore, we evaluated entry of the variants using a VSV-pseudotype entry assay on ACE2 transfected cells [31]. For both human and hamster ACE2, we observed increased entry by Delta over Alpha. For human ACE2, entry by Delta was significantly higher by 1.6-fold over than Alpha (median fold increase relative to no spike = 44.8 and 28.4, respectively, p = 0.0005, N = 8, ordinary two-way ANOVA, followed by Šídák’s multiple comparisons test), while for hamster ACE2, entry was significantly increased by 1.2-fold (median = 59.3 and 47.8, respectively, p = 0.005) (Figure 1D). This suggests that the Syrian hamster model should recapitulate the entry-specific competitive advantage of Delta over Alpha observed in humans.

### Detection of infectious SARS-CoV-2 in air samples is limited to first 72h

Syrian hamsters were inoculated with a low dose (10^3^ TCID_50_, intranasal (IN), N = 10 per group) of SARS-COV-2 Delta or Alpha. Animals were monitored for 14 days post inoculation (DPI). We observed no significant differences in weight loss or viral titers in lung or nasal turbinates between the variants (Figure S 1A–C). At 14 DPI, hamsters (N = 5) mounted a robust anti-spike IgG antibody response, and the overall binding pattern was similar between Alpha and Delta (Figure S 1D,E). In a live virus neutralization assay, homologous virus was significantly better neutralized as compared to the heterologous variant (Figure S 1F,G), but no significant difference was determined between the neutralization capacity against the respective homologous variant (median reciprocal virus neutralization titer = 320 (Alpha anti-Alpha)/ 320 (Delta anti-Delta), p = 0.9568, N = 5, ordinary two-way ANOVA, followed by Tukey’s multiple comparisons test).

We determined the window of SARS-CoV-2 shedding for Alpha and Delta using swabs from the upper respiratory tract and air sampling from cages, quantifying virus using gRNA, sgRNA, and infectious virus titers. Oral swabs remained positive for gRNA and sgRNA until 7 days post inoculation (DPI), but infectious virus dropped to undetectable levels after 4 DPI (Figure S 2A). Cage air was sampled during the first 5 days of infection in 24 h time windows, from cages containing 2 or 3 animals, grouped by sex. gRNA and sgRNA were detectable as early as 1 DPI in 50% of air samples and remained high through 5 DPI, while infectious virus was detectable for a shorter window, from 1 to 3 DPI (Figure S 2B).

### Mathematical modeling demonstrates airborne shedding peaks later and declines faster than oral swab viral load

We quantified heterogeneity in shedding by variant, sex, and sampling method by fitting a mathematical model of within-hamster virus kinetics (see SI Mathematical Model Methods) to the data. Virus was detectable and peaked earlier in oral swabs (approximately 24 h post inoculation) than virus sampled from the air (approximately 48 h post inoculation), and quantity of detected virus declined slower in the swabs (Figure 1E,F). gRNA and sgRNA declined slower than infectious virus both in the air and in swabs. In our hamster model, oral swab data was a poor proxy for airborne shedding, even when we directly quantified infectious virus titers. This was due to a lag between peak swab shedding and peak airborne shedding. Inferred within-host exponential growth and decay rates were similar for the two variants. For both variants, males shed more virus than females, even after accounting for males’ higher respiration rates in measurements of shedding into the air. We found a slightly higher ratio of infectious virus to sgRNA in air samples for Delta than for Alpha (Figure 1F, Figure S 2C,D); this likely reflects the higher per-virion infectivity of the Delta variant in the VERO E6 (UNC) cell line used for plaque assays.

We also found substantial individual-level heterogeneity in airborne shedding, even after accounting for sex and variant (Figure 1F). For example, air samples from cage 5 had more than twice as many peak plaques per capita than cage 6, even though both cages contained hamsters of the same sex, inoculated by the same dose, route, and variant. Our model captures this, with substantial inferred heterogeneity in individual airborne shedding in PFU per hour, both in timing and in height of peak (Figure 1F). This observed heterogeneity of airborne shedding may help explain the observed over-dispersion in SARS-CoV-2 transmission in humans: some individuals transmit to many others while other individuals transmit to no one.

### Changes in breathing profile after SARS-CoV-2 infection precede onset of weight loss and are variant and sex-dependent

**Figure 2.**
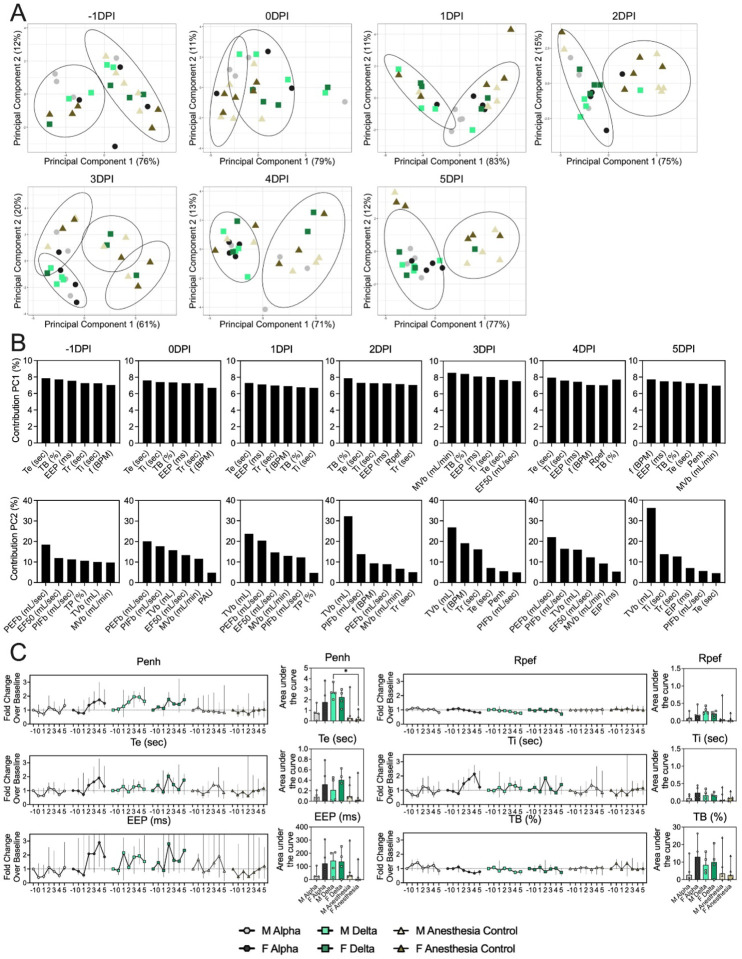
Lung function and breathing changes after SARS-CoV-2 infection with Alpha and Delta. Syrian hamsters were inoculated with 10^2^ TCID_50_ via the intranasal route with Alpha or Delta and respiratory pathology compared on day 5. **A.** Lung function was assessed on day −1, 0, 1, 2, 3, 4 and 5 by whole body plethysmography. Principal component analysis was used to investigate individual variance. Depicted are principal component (PC) 1 and 2 for each day, showing individual animals (colors refer to legend on right, sex-separated) and clusters (black ellipses). **B.** Individual loading plots for contributions of top 6 variables to PC1 and 2 at each day. **C.** Relevant lung function parameters. Line graphs depicting median and 95% CI fold change values (left) and area under the curve (AUC, right). Abbreviations: Expiratory time (Te), inspiratory time (Ti), percentage of breath occupied by the transition from inspiration to expiration (TB), end expiratory pause (EEP), breathing frequency (f), peak inspiratory flow (PIFb), peak expiratory flow (PEFb), tidal volume (TVb), minute volume (MVb), enhanced pause (Penh).

Next, we analyzed the onset of changes in lung function and breathing patterns. Whole body plethysmography (WBP) was performed for the first 5 days after inoculation (Figure S 1A). Expiratory time (Te), inspiratory time (Ti), percentage of breath occupied by the transition from inspiration to expiration (TB), end expiratory pause (EEP), breathing frequency (f), peak inspiratory flow (PIFb), peak expiratory flow (PEFb), tidal volume (TVb), minute volume (MVb) and enhanced pause (Penh) were used to assess changes in pulmonary function throughout infection. Principal component analysis was used to determine trends in lung function changes across all groups (Figure 2A). This revealed a large degree of inherent variation in individual hamster plethysmography measures. Before inoculation there was no discernible pattern to the clustering observed besides a slight separation by sex. Beginning at 2 DPI, we observed a separation of infected and control animals. This coincided with the observation that all SARS-CoV-2 animals visibly decreased activity levels after 2 DPI, reducing exploratory activity and grooming with sporadic short convulsions which may represent coughing. No single parameter had an overwhelming influence on clustering, though several parameters contributed strongly across all days: Te, Ti, TB, EEP, f, PIFb, PEFb, TVb, and MVb (Figure 2 B,C).

Broad patterns emerged by variant and by sex. Cumulative Penh AUC values for all infected groups were increased compared to the sex-matched control hamsters (p = 0.022, Kruskal-Wallis test, N = 5). The median Penh AUC values for Alpha, Delta, and control males were 0.741, 2.666, and 0.163, respectively (p = 0.062). The median Penh AUC values for Alpha females, Delta females, and control females were 1.783, 2.255, and 0.159, respectively (p = 0.019). At 4 DPI, the median fold change Penh values for Alpha males and Delta males were 0.793 and 1.929, respectively, as compared to 0.857 for control males. The corresponding Penh values for Alpha, Delta, and control females were 1.736, 1.410, and 1.008, respectively. The separation on 4 DPI did not translate to significant changes in more traditional measures of respiratory function, including f, TVb, and MVb, but were echoed in Te, Ti, and EEP. However, a significant difference was only found for median Ti values in females at 4 DPI (p = 0.03). In addition, compared to Penh, we observed increased amounts of individual variability for these parameters across all groups. Throughout our analyses, it appeared that Alpha-infected male hamsters more closely resembled the control hamsters than any of the other infected groups. This was not due to variant-linked differences between the underlying relationships between parameters, which showed no differences (Figure S 3A,B). The pathology in nasal turbinates and lungs did not differ significantly between animals (Figure S 4A–O). Pathological changes were consistent with those described previously for COVID-19 in Syrian hamsters after intranasal inoculation with other SARS-CoV-2 strains [22]. These results indicate that the underlying differences between individual hamsters and sexes in baseline respiratory metrics may influence airborne transmission, as these individual differences are retained while infectious virus shedding is highest.

### Changes in exhaled aerosol aerodynamic profile after SARS-CoV-2 infection precede acute disease, are variant and sex-dependent

**Figure 3:**
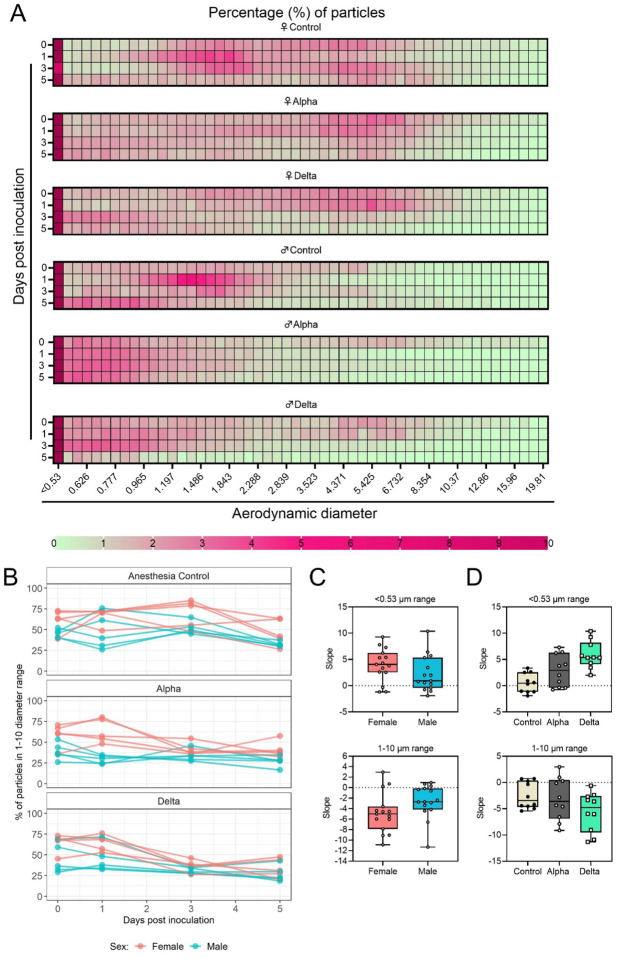
Aerodynamic particle analysis of SARS-CoV-2 infected hamsters. **A.** Syrian hamsters were inoculated with 10^2^ TCID_50_ via the intranasal route with Alpha or Delta. Aerodynamic diameter profile of exhaled particles was analyzed on day 0, 1, 3, and 5. Heatmap shows rounded median percent of total particles across groups, including the anesthesia control group (N = 10, comprising 5 males and 5 females). Colors refer to scale below. **B.** For each animal, line graph of the percent of particles in the 1–10 μm diameter range by variant group and sex indicated by color. **C and D**. Individual slopes for percent of particles in the <0.53 μm range (top) and 1–10 μm range (bottom) are presented by sex and variant group. Boxplots depict median, interquartile range, and range. Multiple linear regression performed for each diameter range with group and sex as predictors, F-statistic (3,26) = 9.47 for <0.53 μm model and F-statistic (3,26) = 2.62 for 1–10 μm model, with Tukey multiple comparison adjustment for the three variant-group comparisons (95% family-wise confidence level). For <0.53 range, Male-Female (estimate = −1.7, standard error = 0.888, two-sided p = 0.0659); Alpha-Control (estimate = 2.41, standard error = 1.09, two-sided p = 0.0874), Delta-Control (estimate = 5.40, standard error = 1.09, two-sided p = 0.0001), Delta-Alpha (estimate = 2.99, standard error = 1.09, two-sided p = 0.0280). For 1–10 range, Male-Female (estimate = 2.19, standard error = 1.23, two-sided p = 0.0875); Alpha-Control (estimate = −0.633, standard error = 1.51, two-sided p = 0.9079), Delta-Control (estimate = −3.098, standard error = 1.51, two-sided p = 0.1197), Delta-Alpha (estimate = −2.465, standard error = 1.51, two-sided p = 0.2498). Grey = Alpha, teal = Delta, beige = anesthesia control, red = female, blue = male.

Over the course of disease, a decrease in physical activity was observed, but it is plausible that decreased behavioral activity and subsequent changes in breathing patterns may also influence the aerodynamic particle profile of exhaled large and fine aerosols. Alpha and Delta inoculated groups (N = 10 each) and a control group (N = 10) were individually evaluated on 0, 1, 3, and 5 DPI. (Figure 3). Across each variant group, particle diameter size <0.53 μm was the most abundant (Figure 3A). No consistent, significant overall change in the number of overall particles across all sizes was observed between groups (Figure S 5C). Particles between 1 and 10 μm in diameter, most relevant for fine aerosol transmission [32], were examined. At baseline (0 DPI), females across all groups produced a higher proportion of droplets in the 1–10 μm diameter range compared to males (Figure 3B). We conducted a separate experiment detailing activity levels of five age-matched uninfected hamsters over 25 minutes total in relation to their particle production profile. When examining the detailed records collected during this experiment, we found that the production of particles in females in the 1–10 μm range appears to be driven by more frequent exploratory behavior in females (Figure S 5B). At 3 DPI, the particle profiles shifted towards smaller aerodynamic diameters in the infected groups. At 5 DPI, even control animals demonstrated reduced exploratory behavior, resulting in a reduction of particles in the 1–10 μm range, which could be due to acclimatization to the chamber. This resulted in an overall shift in particle size from the 1–10 μm range to the <0.53 μm range. To analyze these data, individual slopes for each animal were calculated using simple linear regression across the four timepoints (Percent ~ Intercept + Slope * Day) for percent of particles in the <0.53 μm range and percent of particles in the 1–10 μm range (Figure 3 B,C,D), and multiple linear regression was performed (Figure 3 C,D). Holding variant group constant, the slope for percent of particles in the 1–10 μm range was, on average, 2.2 times higher for males compared to females while the <0.53 μm model estimate was 1.7 times lower (Figure 3C). Comparing variant group while holding sex constant, we found that the Delta group had a steeper decline for percent of particles in the 1–10 μm range (and a steeper incline for <0.53 μm); a similar, but not as steep of a trend was observed for Alpha (Figure 3 C,D). After adjusting for multiple comparisons using Tukey, differences in <0.53 μm slopes were observed for Delta vs. Control (5.4 times higher, two-sided p = 0.0001) and Delta vs. Alpha (3 times higher, two-sided p = 0.0280), and for Alpha vs. Control, 2.4 times higher (two-sided p = 0.0874) (Figure 3 D); for the 1–10 μm model, differences were not as pronounced. This suggests that after SARS-CoV-2 infection, the exhaled droplet profile changes towards the production of fine particles < 1 μm, which are most likely to remain suspended in air for long durations. Interestingly, the data also implies that beyond disease severity [33] and shedding, sex may impact the ability to transmit virus in airborne droplets by impacting both the total number of particles produced in each size bracket, as well as magnitude in shift after infection. A linear mixed model was considered and produced virtually the same results as the simpler analysis described above; the corresponding linear mixed model estimates were the same and standard errors were similar.

### Alpha and Delta VOC attack rates reveal minimal individual risk of dual infection *in-vivo*

**Figure 4.**
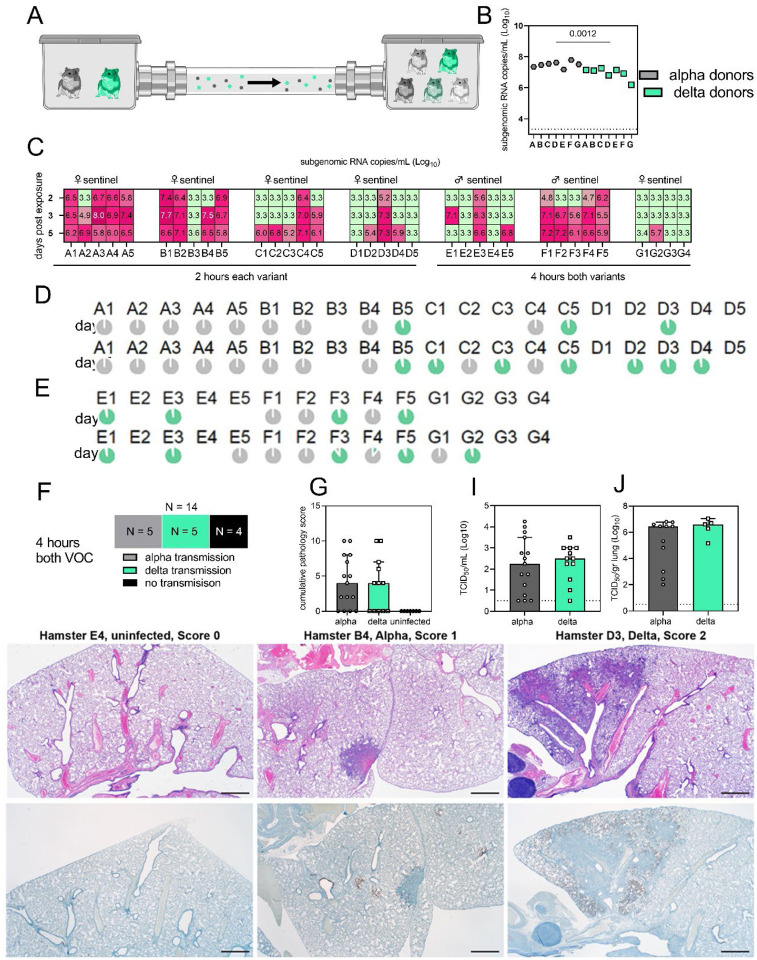
Airborne attack rate of Alpha and Delta SARS-CoV-2 variants. Donor animals (N = 7) were inoculated with either the Alpha or Delta variant with 10^3^ TCID_50_ via the intranasal route and paired together randomly (1:1 ratio) in 7 attack rate scenarios (A-G). To each pair of donors, one day after inoculation, 4–5 sentinels were exposed for a duration of 4 hours (i.e., hours 24–28 post inoculation) in an aerosol transmission set-up at 200 cm distance. **A.** Schematic figure of the transmission set-up. **B**. Day 1 sgRNA detected in oral swabs taken from each donor after exposure ended. Individuals are depicted. Wilcoxon test, N = 7. Grey = Alpha, teal = Delta inoculated donors. **C**. Respiratory shedding measured by viral load in oropharyngeal swabs; measured by sgRNA on day 2, 3, and 5 for each sentinel. Animals are grouped by scenario. Colors refer to legend on right. 3.3 = limit of quality. **D, E.** Percentage of Alpha and Delta detected in oropharyngeal swabs taken at day 2 and day 5 post exposure for each individual determined by deep sequencing. Pie-charts depict individual animals. Grey = Alpha, teal = Delta. **F.** Summarized attack rates for iterations EG. **G**. Cumulative pathology score of sentinels on day 5 post exposure. Bar-chart depicting median, 96% CI, and individuals, Mann-Whitney test. **H.** Lung histologic lesion scores 0, 1, 2. Score 0, Normal lung devoid of immunoreactivity. Score 1, A solitary focus of inflammation (circled) surrounded by normal lung. Bronchiole (*) and alveolar (>) immunoreactivity. Score 2, Multiple foci of coalescing inflammation centered on airways. Immunoreactive alveoli throughout the left half of the image. 20x HE (top row) and anti-SARS-2 IHC (bottom row). **I, J**. Viral load measured via infectious virus titer in swabs and lungs of sentinels on day 5. Bar-chart depicting median, 96% CI, and individuals, Mann-Whitney test. Grey = Alpha, teal = Delta, p-values indicated where significant. Abbreviations: TCID, Tissue Culture Infectious Dose.

We next compared attack rates between Alpha and Delta during a 4 h exposure window at 200 cm distance. Groups of sentinels (N = 4 or 5) were exposed to two Donor animals, one inoculated with Alpha and one inoculated with Delta (Figure 4A). Donors were IN inoculated with 10^3^ TCID_50_. sgRNA in oral swabs taken on 1 DPI varied between animals (Figure 4B). Sentinels were either exposed first for 2 h to one variant and then for 2 h to the second (Figure 4C, first 4 iterations), or to both variants at the same time for 4 h (last three iterations). Transmission was confirmed by sgRNA in oral swabs collected from all sentinels at 2, 3, and 5 DPE. On 2 DPE, N = 13/34 sentinels were positive for sgRNA in oral swabs, N = 19/34 on 3 DPE and N = 27/34 on 5 DPE. Swabs from 3 DPE and 5 DPE were sequenced, and the percentage of reads mapped to Alpha, and Delta were compared (Figure 4 D,E).

All animals had only one variant detectable on day 3. In total, 12 sentinels were infected with Alpha and 7 with Delta by 3 DPE. At 5 DPE, slightly more sentinels shed Alpha (N = 15 vs. N = 12). Interestingly, we observed one superspreading event in iteration A, in which one donor animal transmitted Alpha to all sentinels. For all other iterations, either both donors managed to transmit to at least one sentinel, or not all sentinels were infected. For the iterations with simultaneous exposure, attack rates were similar and statistically indistinguishable: Alpha = 50 %, Delta = 42.8 % (two-tailed Fisher’s exact test, p = 1) (Figure 4F). In one simultaneous exposure (iteration F), three sentinels had both Delta and Alpha detectable at 5 DPE. In two Delta was dominant, and in one Alpha, always with the other variant in the clear minority (<15%). We did not observe any other such coinfections (defined as a PCR positive animal with both Alpha and Delta at 5% frequency or higher by NGS). This led us to ask whether there was virus interference in sequential exposures - that is, whether established infection with one variant could reduce the probability of successful infection given a later exposure.

To assess this, we used our within-host dynamics model to calculate the estimated infection probabilities for Alpha and Delta for each sentinel in each iteration, assuming each sentinel is exposed independently, but accounting for the different exposure durations, donor sexes, and donor viral load (as measured by oral swabs). From those probabilities, we then calculated posterior probability distributions for the number of co-infections predicted to occur in each iteration if Alpha and Delta infections occurred independently and did not interfere with each other (SI Mathematical Model Methods, Figures S1–3). We found that our observed coinfections were consistent with this null model; our data do not provide clear evidence of virus interference during sequential exposure, though they also do not rule out such an effect.

Next, we investigated whether the different variants would impact sentinels differently. In general, all sentinels at 5 DPE demonstrated “no” to “minimal” to “mild” lesions affecting no more than 25% of the lungs (Figure 4H). No significant differences in cumulative pathology were observed between Alpha or Delta variant infected sentinels (Figure 4G). Immunoreactivity was lower overall compared to IN inoculated animals. No significant differences in infectious virus titer were found on 5 DPE in oral swabs (Alpha: median 2.25 TCID_50_ (Log_10_) and Delta: median 2.5 TCID_50_ (Log_10_), p = 0.7452, Mann-Whitney test) or lungs (Alpha: median 6.4 TCID_50_ (Log_10_) and Delta: median 6.6 TCID_50_ (Log_10_), p = 0.2674) (Figure 4I,J). These data imply that in the Syrian hamster model, both Alpha and Delta VOCs demonstrate similarly high attack rates in a fine aerosol transmission set-up and neither variant has a direct competitive advantage after transmission. It also shows that dual infections during simultaneous or closely adjacent exposure windows are less likely than expected if the infections occurred independently.

### Limited sustainability of heterologous VOC populations through multiple rounds of airborne transmission

**Figure 5.**
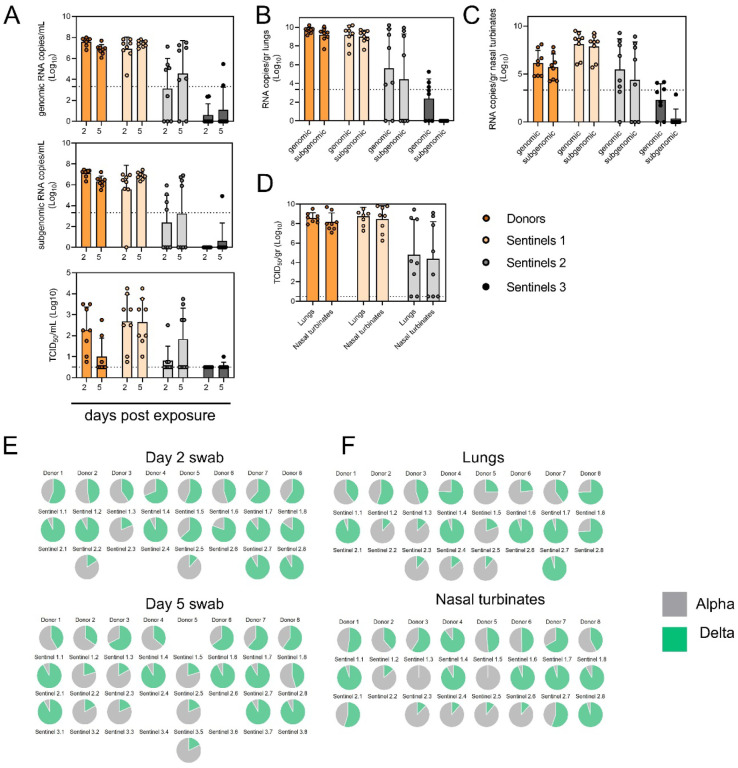
Airborne competitiveness of Alpha and Delta SARS-CoV-2 variants. Donor animals (N = 8) were inoculated with Alpha and Delta variant with 5 × 10^2^ TCID_50_, respectively, via the intranasal route (1:1 ratio), and three groups of sentinels (Sentinels 1, 2, and 3) were exposed subsequently at a 16.5 cm distance. Animals were exposed at a 1:1 ratio; exposure occurred on day 1 (Donors → Sentinels 1) and day 2 (Sentinels → Sentinels) and lasted for 24 hours for each chain link. **A.** Respiratory shedding measured by viral load in oropharyngeal swabs; measured by gRNA, sgRNA, and infectious titers on days 2 and day 5 post exposure. Bar-chart depicting median, 96% CI and individuals, N = 8, ordinary two-way ANOVA followed by Šídák’s multiple comparisons test. **B, C, D.** Corresponding gRNA, sgRNA, and infectious virus in lungs and nasal turbinates sampled five days post exposure, measured by titration. Bar-chart depicting median, 96% CI and individuals, N = 8, ordinary two-way ANOVA, followed by Šídák’s multiple comparisons test. Dark orange = Donors, light orange = Sentinels 1, grey = Sentinels 2, dark grey = Sentinels 3, p-values indicated where significant. Dotted line = limit of quality. **E.** Percentage of Alpha and Delta detected in oropharyngeal swabs taken at days 2 and day 5 post exposure for each individual donor and sentinel, determined by deep sequencing. Pie-charts depict individual animals. Grey = Alpha, teal = Delta. **F.** Lung and nasal turbinate samples. Abbreviations: TCID, Tissue Culture Infectious Dose.

To assess the transmission efficiency in direct competition between the Alpha and Delta VOCs, we conducted an airborne transmission experiment over three subsequent rounds of exposure [23]. Donor animals (N = 8) were inoculated IN with 5 × 10^2^ TCID_50_ of SARS-CoV-2 variants Alpha and Delta (1:1 mixture) and eight sentinels were exposed (Sentinels 1, 1:1 ratio) on 1 DPI for 24 h (first chain link, exposure window: 24–48 h post inoculation of the donors) (Figure 5). Two days after the start of this exposure, the eight sentinels were placed into the donor side of a new cage and eight new sentinels (Sentinels 2)2 were exposed for 24 hours (second chain link, exposure window 48–72 h post exposure start of the Sentinels 1). Again, 2 days after exposure start, this sequence was repeated for Sentinels 3 (third chain link, exposure window 48–72 h post exposure start of the Sentinels 2). All animals were individually housed between exposures, and after exposure as well for the sentinels. We assessed viral presence in oropharyngeal swabs taken from all animals at 2 and 5 DPI/DPE. While all Sentinels 1 demonstrated active shedding at 2 and 5 DPE, in the Sentinels 2 group no viral RNA was detected in 2/8 animals and no infectious virus in 4/8 by 5 DPE. In the Sentinels 3 group, sgRNA and infectious virus were only detected robustly in one animal on 5 DPE. In contrast to donor animals, all infected sentinels exhibited higher shedding on day 5 compared to day 2 (2 DPI / 5 DPI Donors: median gRNA = 7.8 / 6.9 copies/mL (Log^10^), median sgRNA = 7.2 / 6.4 copies/mL (Log_10_)), median infectious virus titer = 2.3 / 0.5 TCID_50_/mL (Log_10_); Sentinels 1 (median gRNA = 7.2 / 7.4 copies/mL (Log_10_), median sgRNA = 6.4 / 6.9 copies/mL (Log_10_), median infectious virus titer = 2.9 / 2.6 TCID_50_/mL (Log_10_); Sentinels 2 = median gRNA = 3.7 / 5.4 copies/mL (Log_10_), median sgRNA = 1.8 / 3.0 copies/mL (Log_10_), median infectious virus titer = 0.5 / 1.6 TCID_50_/mL (Log_10_)) (Figure 5A). Taken together, this evidence suggests that the infectious shedding profile shifts later and decreases in magnitude with successive generations of transmission. This could be explained by lower exposure doses causing lower and slower infections in the recipients.

We then proceeded to compare the viral loads in the lungs and nasal turbinates at 5 DPE. Viral gRNA was detected in the lungs (Figure 5B) and nasal turbinates (Figure 5C) of all Donors (lungs: median gRNA = 9.7 copies/gr tissue (Log_10_), nasal turbinates: median gRNA = 6.2 copies/gr tissue (Log_10_)). Interestingly, while the gRNA amount was similar in lungs between Donors and Sentinels 1 (lungs: median gRNA = 9.5 copies/gr tissue (Log_10_)), it was increased in nasal turbinates for the Sentinel 1 group (nasal turbinates: median gRNA = 8.6 copies/gr tissue (Log_10_)). Similarly, sgRNA was increased in Sentinels 1 as compared to Donors in nasal turbinates, but not lungs (Donors = lungs: median sgRNA = 9.4 copies/gr tissue (Log_10_), nasal turbinates: median sgRNA = 5.7 copies/gr tissue (Log10); Sentinels 1 = lungs: median sgRNA = 9.2 copies/gr tissue (Log_10_), nasal turbinates: median sgRNA = 8.4 copies/gr tissue (Log_10_)). Viral gRNA above the level of quantification was detectable in 6/8 Sentinels 2 in both lungs and nasal turbinates, yet sgRNA was only detected in 4/8 Sentinels 2 in lungs and 5/8 in nasal turbinates. Even though gRNA was detected in 3/8 Sentinels 3, no animal had detectable sgRNA in either lungs or nasal turbinates, signaling a lack of active virus replication. To confirm this, infectious virus was analyzed in both tissues for the Donors, Sentinels 1, and Sentinels 2 groups. In both tissues titers were marginally higher in Sentinels 1 (median TCID_50_ / gr tissue (Log_10_) Donors: lungs = 8.6, nasal turbinates = 8.0; Sentinels 1: lungs = 8.9, nasal turbinates = 8.8). Infectious virus was present in 6/8 Sentinels 2 in lungs and 5/8 in nasal turbinates.

Hence, even though the exposure interval for the second and third chain links were started 48 hours after the start of their own exposure, not all Sentinels 2 became infected, and only one Sentinel 3 animal became infected and demonstrated shedding. We conducted a separate experiment to assess viral loads in the respiratory tract after SARS-CoV-2 airborne transmission at 2 DPI/DPE. While infectious virus was present in oral swabs from all sentinels, virus in lungs and nasal turbinates was not present in all animals (Figure S 6). This suggests that replication in the oropharyngeal cavity precedes replication in other compartments of the respiratory tract but that (as shown in Figure 1E,F) oral swabs may not be a reliable indicator of release of infectious virus into the air. Even though lung viral titers were similar between animals that became infected, gross lung pathology and lung weights decreased with each transmission chain link as previously described (Figure S 7A,B) [23].

To determine the competitiveness of the variants, we analyzed the relative composition of the two viruses using next generation sequencing (Figure 5 E,F, Table S1). Neither variant significantly out-competed the other. We first compared the percentage of Delta in oral swabs taken on 2 DPI/DPE, the day of exposure of the next chain link. In Donors, no variant was more prevalent across animals or clearly outcompeted the other within one host (median = 56.5% Delta, range = 40.3–69%). After the first transmission event, Delta outcompeted Alpha at 2 DPE (median = 87.3% Delta, range = 19–92.7%), while after the second transmission event, half (N = 2/4) the animals shed either > 80% either Alpha or Delta. Notably, and in strong contrast to the dual donor experiments described above, every sentinel animal exhibited a mixed infection at 2 DPE, often with proportions resembling those in the donor. This indicates that the transmission bottleneck for these 24 h exposures is not narrow, and viral diversity is transmitting between hosts.

Next, we looked at the selective pressure within the host. By 5 DPI/DPE, no clear difference was observed in Donors (median = 60 % Delta, range = 34.3–67.7%), but in the Sentinels 1 group Alpha overtook Delta in three animals (median = 68.3 % Delta, range = 17–92.3%), while the reverse was never seen. In one animal, we observed a balanced infection established between both variants at 5 DPE (Sentinel 1.8). In the Sentinels 2 group, Alpha was the dominant variant in N = 3/8 animals, and Delta dominated in 3/8 (median = 55% Delta, range = 17–92.7%). The one Sentinel 3 animal for which transmission occurred shed nearly exclusively Alpha. This suggests that within one host, Alpha was marginally more successful at outcompeting Delta in the oropharyngeal cavity.

We then assessed virus sequences in lungs and nasal turbinates to understand if the selective pressure on both variants is influenced by spatial dynamics. In Donor lungs, the percentage of Alpha was marginally higher on 5 DPI (median = 42.3% Delta, range = 23.3–75.7%). In the Sentinels groups, either Alpha or Delta outcompeted the other variant within each animal, only one animal (Sentinel 1.8) revealing both variants > 15%. In N = 5/8 Sentinels 1, yet only in N = 1/4 Sentinel 2 animals, Delta outcompeted Alpha. Sequencing of virus isolated from nasal turbinates reproduced this pattern. In Donors, neither variant demonstrated a completive advantage (median = 51.2% Delta, range = 38.7–89.3%). In N = 5/8 Sentinels 1, and N = 3/8 Sentinels 2, Delta outcompeted Alpha. Combined, a trend, while not significant, was observed for increased replication of Delta after the first transmission event, but not after the second, and in the oropharyngeal cavity (swabs) as opposed to lungs. Combined, these data suggest that Delta may transmit marginally better via the airborne route but once established in the animal, Alpha is more fit.

## Discussion

In immunologically naïve humans, peak SARS-CoV-2 shedding is reached multiple days after exposure and occurs multiple days before onset of symptoms [34]. It is not known how this informs the window of transmissibility, which is poorly understood and difficult to study in the absence of controlled exposures. Given the predominance of airborne transmission in driving the pandemic, measuring the quantity of exhaled virus and size distribution of airborne particles is more likely to provide insight into the window of transmissibility than simply measuring infectious virus in the upper respiratory tract. In addition, the shedding of virus in large and fine aerosols may be a function of physiological changes after infection. Such questions are more readily addressed in an experimental model for transmission, such as the Syrian hamster, than in humans. For hamsters inoculated with wildtype and Alpha VOC SARS-CoV-2, past studies have shown that transmissibility is limited to the first three days after inoculation. This coincided with peak shedding and ends before the onset of weight loss, clinical manifestation, and loss of infectious virus shedding in the upper respiratory tract [2, 21, 35]. We set out to determine if infection with VOCs Alpha and Delta affects host-derived determinants of airborne transmission efficiency early after infection, which may explain this restriction.

Based on our data, we inferred that the overall phenotypic properties of Alpha and Delta in the hamster model were similar. As with other viral infections, COVID-19 severity is increased for the male sex as a consequence of sex-dependent immunity, genetics, or co-morbidities [39, 40]. Previous work in Syrian hamsters has found that male sex was linked to increased disease severity, virus replication, and possibly an increase in measured susceptibility through the intranasal route [30]. We found higher peak virus and duration of shedding in male hamsters compared to females, but with substantial inter-individual variation. Human studies have found similar peak viral RNA levels for Alpha and Delta [36, 37] despite suggested epidemiological differences in humans, including Delta’s higher transmissibility [38], shorter generation interval [39], and greater risk of severe disease [40]. Our results place these incongruous observational findings in context; we observe similar kinetics in a controlled setting with experimentally infected and sampled hamsters, both within host and in oropharyngeal swabs samples taken to measure upper respiratory shedding.

We confirm that viral RNA levels can be a poor proxy for infectious virus, especially later in infection, as also seen in humans [41]. We also show that neither oral swab sgRNA nor swab infectious virus, while nominally better, are a particularly good proxy for virus sampled from air. In particular, inferred swab peaks occurred approximately 24 h post-infection, while inferred air shedding peaks occurred a day later, at approximately 48 h post-infection. Attempts to quantify an individual’s airborne infectiousness from swab measurements - even of infectious virus titer - should thus be interpreted with caution. Similarly, viral load should not be treated as a single quantity that rises and falls synchronously throughout the host; spatial models of infection may be required to identify the best correlates of infectiousness [42]. Crucially, there is a period early in infection (around 24 h post-infection in inoculated hamsters) when oral swabs show high viral titers, but air samples exhibit low or undetectable levels of virus. It is possible that during this time window the physiochemical properties of the mucus change and limit aerosol generation [32]. Our data suggest that oropharyngeal swab-based comparisons of variant viral loads - even those that measure infectious virus rather than viral RNA - should be interpreted with caution in terms of their relevance for transmission potential.

While past studies have used whole body plethysmography to differentiate the impact of VOCs on lung function, these have mostly focused on using mathematically-derived parameters such as Penh, to compare significant differences on pathology in late acute infection [26]. We set out to delineate the parameters changing more comprehensively over time. Even before challenge, we observed high individual noise in the hamster population and stark differences by behavioral state which correlated with sex, highlighting that future studies of this nature may require increased acclimatization of the animals to the experimental procedures. Yet even taking this caveat into consideration, we observed that for both variants changes in breathing patterns were observable as early as 2 DPI, preceding observable clinical symptoms and acute pathology, but coinciding with the window of time when infectious virus was found in the air. Previous work in the Syrian hamster model found that the majority of virus was contained within droplet nuclei <5 μm in size [27]. We report a rise in <0.53 μm particles and a drop in particles in the 1–10 μm range. We observed that the reduction in percent of droplets in the 1–10 μm range was more pronounced in females compared to males, and a steeper decline was observed after Delta inoculation compared to Alpha. One of the caveats of these measurements in small animals is that detected particles may come from aerosolized fomites, and residual dust generated by movement [43]. In our system, we did not detect any particles originating from dead animals or the environment, but we also saw a noticeable reduction of particles across sizes when movement was minimal, or animals were deeply asleep. This state would occur more frequently during acute disease, which confounds if the reduction in particles is due to reduced behavior or is physiological in nature. Considering the noise and individual variability in the lung function data we collected, we did not observe that this shift in particle production was accompanied by a consistent change in either breathing frequency, tidal volume, or minute volume.

Symptomatic humans with COVID-19 have been shown to exhale fewer particles than uninfected individuals during normal breathing, but not during coughing [44]. Talking or singing, behaviors which are not modelled in the Syrian hamster, have also been implicated as a source of virus-loaded aerosols [12]. Specifically fine aerosols have been found to be the major source of virus-loaded droplets. One study in humans found viral RNA in 45% of fine (≤ 5 μm) and 31% of coarse (>5 μm) aerosols sampled from patients [45], while a second study confirmed that amongst all detectable RNA sampled while talking or singing, 85% was detected in fine aerosols [14]. This suggests that our observation of a shorter duration of measurable infectious virus in air, as opposed to the upper respiratory tract, could be partially due to early changes in airway constriction and a reduction in exhaled particles of the optimal size range for transmission and virus aerosol stability. The mechanisms involved in the changing aerodynamic particle profile, and the distribution of viral RNA across particle sizes, require further characterization in the Syrian hamster. Our data does not corroborate findings in non-human primates, where one study reported an increased total number of exhaled particles in SARS-CoV-2 infected animals [15]. This could be due to discrepancies in methodology and sensitivity recording particles in the <1 μm range, or it could reflect species-specific differences, and requires further work to understand.

Lastly, we compared the transmission efficiency of the Alpha and Delta variants in this system. We did not find a clear transmission advantage for Delta over Alpha in Syrian hamsters, in either an attack rate simulation or when comparing intra- and inter-host competitiveness over multiple generations of airborne transmission. This contrasts sharply with epidemiological observations in the human population, where Delta rapidly swept through populations where Alpha (or other VOCs) had been circulating. This highlights the fact that the Syrian hamster model may not completely recapitulate all aspects of SARS-CoV-2 virus kinetics and transmission in humans, particularly as the virus continues to adapt to its human host. Given that with the emergence of Delta, a large part of the human population was already previously exposed to and/or vaccinated against SARS-CoV-2, the underlying immune status might have played a role in some geographical regions. Interestingly, analyses of the cross-neutralization between Alpha and Delta suggest subtly different antigenic profiles [46].

Our experiments enabled us to study the competition between variants in different transmission contexts. We hypothesized that two variants with similar replication within host, as we have described here for Alpha and Delta, would be more likely to be passed on during an airborne transmission event as a mixed-variant population. We previously demonstrated that this is highly unlikely when one variant outcompeted the other (Lineage A and Alpha), because the intrinsic phenotypic advantages of Alpha strongly favored this variant over the competitor [2, 47]. Interestingly, our two transmission experiments yielded different outcomes. Using our setup with sentinel hamsters sequentially or simultaneously exposed to two donor hamsters individually infected with either Alpha or Delta, we did not observe any dual infection in all but three sentinels, which belonged to the same simultaneous exposure iteration, and all had one dominant and one minority variant in 5 DPE oral swabs. We observed one superspreading event, in which a male donor infected with Alpha infected all five of the sentinels. In all other cages, some sentinels were uninfected or infected by the other donor. In contrast, when we exposed hamsters to donors infected with both Alpha and Delta, these mixed-variant populations were passed onwards. Dual infections were not retained equally, and virus frequencies shifted within each animal, indicating that in all investigated compartments of the respiratory tract, additional factors such as the innate immune response impose selective pressure on the virus populations.

Taken together, our findings raise important questions regarding the nature of transmission as “single-virion” events as opposed to respiratory particles carrying viral populations of multiple virions. Our data imply that when exposures are relatively brief, the risk of multiple infections (and hence, the risk of recombination) may be relatively low on the individual level. In the back-to-back sequential exposures to two donors, we tended to see 0 (no infection) or 1 hit (only one variant) in all sentinels. The minority variants seen in a few sentinels only after simultaneous 4 h exposure to the two variants show that it is possible to have a second ‘single-hit’ within a short time frame. For both exposure setups, the observed number of dual infections is indistinguishable from what is expected if these infection events occur independently. When a second hit does occur, the virus has a delayed start relative to the first infecting virus, and therefore is only detectable as a minority variant after a few days have gone by. In contrast, after 24 h exposures we saw many hits (at least two virions and probably more, as both variants were present with a range of frequencies at 2 DPE). This requires the assumption that the target tissues in the respiratory tract allowed multiple initial infections to occur, and that the innate antiviral responses did not prevent this. Alternatively, if we assume that hits are rare even in a 24 h window, or that the antiviral response in fact does prevent multiple infection events from occurring, our data then suggest that a single donor with mixed infection can send plumes of both variants - and maybe even airborne particles carrying both variants - to infect the sentinel.

Comparing Alpha and Delta, dual infections remained more stable within each host and were passed onwards. This prolongs the time window in which recombination could occur. We speculate that the risk of recombination after a dual infection could be increased if variants possess phenotypic attributes allowing a modicum of simultaneous interhost replication for both before one out-competes the other. Lastly, some newly recombined variants could possess advantageous features for increased transmissibility, but this is not a given [48, 49].

In summary, we observed heterogeneous shedding in air samples, individual variability and sex differences in the droplet profiles, and early changes in breathing patterns in infected animals. These differences among donor hosts may contribute to the explanation for SARS-CoV-2’s over-dispersed transmission pattern. SARS-CoV-2 transmission is characterized by many dead-end infections but also rare high-impact superspreading events. These findings also support that quantitative assessment and control of exhaled aerosols may be critical to slowing the pandemic.

## Materials and Methods

### Ethics Statement

All animal experiments were conducted in an AAALAC International-accredited facility and were approved by the Rocky Mountain Laboratories Institutional Care and Use Committee following the guidelines put forth in the Guide for the Care and Use of Laboratory Animals 8th edition, the Animal Welfare Act, United States Department of Agriculture and the United States Public Health Service Policy on the Humane Care and Use of Laboratory Animals. Protocol number 2021–034-E. Work with infectious SARS-CoV-2 virus strains under BSL3 conditions was approved by the Institutional Biosafety Committee (IBC). For the removal of specimens from high containment areas virus inactivation of all samples was performed according to IBC-approved standard operating procedures.

### Cells and viruses

SARS-CoV-2 variant Alpha (B.1.1.7) (hCoV320 19/England/204820464/2020, EPI_ISL_683466) was obtained from Public Health England via BEI Resources. Variant Delta (B.1.617.2/) (hCoV-19/USA/KY-CDC-2–4242084/2021, EPI_ISL_1823618) was obtained from BEI Resources. Virus propagation was performed in VeroE6 cells in DMEM supplemented with 2% fetal bovine serum, 1 mM L-glutamine, 50 U/mL penicillin and 50 μg/mL streptomycin (DMEM2). VeroE6 cells were maintained in DMEM supplemented with 10% fetal bovine serum, 1 mM L- glutamine, 50 U/mL penicillin and 50 μg/ml streptomycin. No mycoplasma and no contaminants were detected. All virus stocks were sequenced; no SNPs compared to the patient sample sequence were detected in the Delta stock. In the Alpha stock we detected: ORF1AB D3725G: 13% ORF1AB L3826F: 18%.

### Pseudotype entry assay

The spike coding sequences for SARS-CoV-2 variant Alpha and Delta were truncated by deleting 19 aa at the C-terminus. The S proteins with the 19 aa deletions of coronaviruses were previously reported to show increased efficiency incorporating into virions of VSV [50, 51]. These sequences were codon optimized for human cells, then appended with a 5′ kozak expression sequence (GCCACC) and 3′ tetra-glycine linker followed by nucleotides encoding a FLAG-tag sequence (DYKDDDDK). These spike sequences were synthesized and cloned into pcDNA3.1^+^(GenScript). Human and hamster ACE2 (Q9BYF1.2 and GQ262794.1, respectively) were synthesized and cloned into pcDNA3.1^+^ (GenScript). All DNA constructs were verified by Sanger sequencing (ACGT). BHK cells were seeded in black 96-well plates and transfected the next day with 100 ng plasmid DNA encoding human or hamster ACE2, using polyethylenimine (Polysciences). All downstream experiments were performed 24 h post-transfection. Pseudotype production was carried out as described previously [31]. Briefly, plates pre-coated with poly-L-lysine (Sigma–Aldrich) were seeded with 293T cells and transfected the following day with 1,200 ng of empty plasmid and 400 ng of plasmid encoding coronavirus spike or no-spike plasmid control (green fluorescent protein (GFP)). After 24 h, transfected cells were infected with VSVΔG seed particles pseudotyped with VSV-G as previously described [31, 52]. After one hour of incubating with intermittent shaking at 37 °C, cells were washed four times and incubated in 2 mL DMEM supplemented with 2% FBS, penicillin/streptomycin, and L-glutamine for 48 h. Supernatants were collected, centrifuged at 500×*g* for 5 min, aliquoted, and stored at −80 °C. BHK cells previously transfected with ACE2 plasmids of interest were inoculated with equivalent volumes of pseudotype stocks. Plates were then centrifuged at 1200×*g* at 4 °C for one hour and incubated overnight at 37 °C. Approximately 18–20 h post-infection, Bright-Glo luciferase reagent (Promega) was added to each well, 1:1, and luciferase was measured. Relative entry was calculated by normalizing the relative light unit for spike pseudotypes to the plate relative light unit average for the no-spike control. Each figure shows the data for two technical replicates.

### Structural interaction analysis

The locations of the described spike mutations in the Alpha and Delta VOCs were highlighted on the SARS-CoV-2 spike structure (PDB 6ZGE, [53]). To visualize the molecular interactions at the RBD-ACE2 binding interface, the crystal structure of the Alpha variant RBD and human ACE2 complex (PDB 7EKF [29]) was utilized. All figures were generated using The PyMOL Molecular Graphics System (https://www.schrodinger.com/pymol).

### Hamster infection with Alpha and Delta

Four-to-six-week-old female and male Syrian hamsters (ENVIGO) were inoculated (10 animals per virus) intranasally (IN) with either SARS-CoV-2 strain England/204820464/2020 (B.1.1.7/Alpha, EPI_ISL_683466), hCoV-19/USA/KY-CDC-2–4242084/2021 (B.1.617.2/Delta, EPI_ISL_1823618), or no virus (anesthesia controls). IN inoculation was performed with 40 μL sterile DMEM containing 10^3^ TCID_50_ SARS-CoV-2 or simply sterile DMEM. At five days post inoculation (DPI), five hamsters from each route were euthanized and tissues were collected. The remaining 5 animals from each route were euthanized at 14 DPI for disease course assessment and shedding analysis. For the control group, no day 5 necropsy was performed. Hamsters were weighed daily, and oropharyngeal swabs were taken on day 1, 2, 3, and 5. Swabs were collected in 1 mL DMEM with 200 U/mL penicillin and 200 μg/mL streptomycin. For the control group, mock swabs were performed to ensure animals underwent the same anesthesia protocols as infection groups. On day −1, 0, 1, 2, 3, 4, 5, 6, 7, and 14 whole body plethysmography was performed. Profiles of particles produced by hamsters were collected on day 0, 1, 3, and 5. Cage air was sampled on day 0, 1, 2, 3, 4, and 5. Hamsters were observed daily for clinical signs of disease. Necropsies and tissue sampling were performed according to IBC-approved protocols.

### Aerosol cages

Aerosol cages as described by Port *et al*. [23] were used for transmission experiments and air sampling as indicated.

### Air sampling of hamster cages

During the first 5 days, hamsters were housed in modified cages hooked up to an air pump. Air flow was generated at 30 cage changes/h. Between the cage and the pump a 47 mm gelatin air filter was installed. Filters were exchanged in 24-h intervals. The filters were dissolved in 5 mL of DMEM containing 10% FBS and presence of virus was determined by qRT PCR and plaque assay.

### Aerodynamic particle sizing of exhaled droplets

Two strategies were used to measure the aerodynamic diameter of droplets exhaled by hamsters. SARS-CoV-2 inoculated hamsters or uninfected animals were placed into a 1.25 L isoflurane chamber. This allowed free movement of the animal in the chamber. The chamber was hooked up with one port to a HEPA filter. The second port was hooked up to a Model 3321 aerodynamic particle sizer spectrometer (TSI). Both chamber and particle sizer were placed into a BSC class II cabinet. Animals remained in the chamber for 5 × 1 min readings. For each set of readings, there were 52 different particle sizes. For each hamster and timepoint, the total number of particles was calculated (Figure S 5C) and the percent of particles in a particular diameter range was derived using this total. RStudio 2021.09.1 Build 372 Ghost Orchid Release, R version 4.1.2 (2021–11-01), Tidyverse R package version 1.3.1 (2021–04-15), and Emmeans R package version 1.7.2 (2022–01-04) were used for the aerodynamic particle size analysis.

To differentiate between particle profiles produced by an awake and moving animal and those produced by a sleeping animal with limited movement, uninfected age-matched hamsters (3 males and 2 female) were acclimatized to being inside a 38.1 mm inside diameter tube hooked up to a particle sizer (Supplemental Figure 5 A). Both tube and particle sizer were placed into a BSC class II cabinet. To acclimate the animals to the tube, sunflower seeds were provided to encourage investigation and free entry and exit from the tube. After animals became used to being in the tube, ends were capped as depicted and 5 × 5 min readings were taken. The particle size was measured using a Model 3321 aerodynamic particle sizer spectrometer (TSI). Particle size profiles were analyzed using TSI software. As a control, particles originating from empty enclosures and euthanized animals were recorded and found to be absent.

### Whole body plethysmography

Whole body plethysmography was performed on SARS-CoV-2 and uninfected Syrian hamsters (Figure S 1). Animals were individually acclimated to the plethysmography chamber (Buxco Electronics Ltd., NY, USA) for 20 minutes, followed by a 5-minute measurement period with measurements taken continuously and averaged over two-second intervals. Initial data was found to have an especially high rejection index (Rinx) for breaths, so was reanalyzed using a custom Buxco formula to account for differences between mice and hamsters. This included expanding the acceptable balance range, the percent change in volume between inhalation and exhalation, from 20–180% to 15–360%. Reanalysis using this algorithm resulted in the Rinx across all hamsters from one day before infection to 5 days post-infection decreasing from 62.97% to 48.65%. The reanalyzed data were then used for further analysis. Each hamster’s individual averages one day prior to infection were used as their baselines for data analysis.

Areas under the curve (AUCs) for each parameter were calculated for each individual hamster based on their raw deviation from baseline at each time point. Either positive or negative peaks were assessed based on parameter-specific changes. Principal component analyses (PCAs) to visualize any potential clustering of animals over the course of infection were performed for each day on raw values for each of the parameters to accurately capture the true clustering with the least amount of data manipulation. PCAs and associated visualizations were coded in R using RStudio version 1.4.1717 (RStudio Team, 2021). The readxl package version 1.3.1 was then used to import Excel data into RStudio for analysis (Wickham and Bryan, 2019). Only parameters that encapsulated measures of respiratory function were included (zero-centered, scaled). The factoextra package version 1.0.1 (Kassambara and Mundt, 2020) was used to determine the optimal number of clusters for each PCA via the average silhouette width method and results were visualized using the ggplot2 package (Wickham, 2016). Correlation plots were generated based on raw values for each lung function parameter using the corrplot package version 0.90 (Wei and Simko, 2021). The color palette for correlation plots was determined using RColorBrewer version 1.1–2 (Neuwirth, 2014).

### Aerosol transmission attack rate experiment

Four-to-six-week-old female and male Syrian hamsters (ENVIGO) were used. In this experiment naïve hamsters (sentinels) were exposed to donors infected with either Alpha or Delta in the same aerosol transmission set-up to evaluate the attack rates of both variants. Donor hamsters were infected intranasally as described above with 10^3^ TCID_50_ SARS-CoV-2 (Alpha or Delta, N = 7, respectively) and housed individually. After 24 h donor animals were placed into the donor cage. 4 or 5 sentinels were placed into the sentinel cage (N = 34, 7 iterations, **Figure 6 A**), which was connected to the donor cage by a 2 m tube and exposed for 4 h. Air flow was generated between the cages from the donor to the sentinel cage at 30 cage changes/h. One donor inoculated with Alpha, and one donor inoculated with Delta were randomly chosen for each scenario. Both donors were either placed together into the donor cage, or, alternatively, first one donor was placed into the cage for 2 h, then the other for 2 h. To ensure no cross-contamination, the donor cages and the sentinel cages were never opened at the same time, sentinel hamsters were not exposed to the same handling equipment as donors, and equipment was disinfected with either 70% ETOH or 5% Microchem after each sentinel. Regular bedding was replaced by alpha-dri bedding to avoid the generation of dust particles. Oropharyngeal swabs were taken for donors after completion of the exposure and for sentinels on days 2, 3, and 5 after exposure. Swabs were collected in 1 mL DMEM with 200 U/mL penicillin and 200 μg/mL streptomycin. Donors were euthanized after exposure ended, and sentinels were euthanized on day 5 for collection of lungs. All animals were always single-housed outside the exposure window.

### Variant competitiveness transmission chain

Four-to six-week-old female and male Syrian hamsters (ENVIGO) were used. Donor hamsters (N = 8) were infected intranasally as described above with 10^3^ TCID_50_ SARS-CoV-2 at a 1:1 ratio of Alpha and Delta (exact titration of the inoculum for both variants = 503 TCID_50_, 80% Delta sequencing reads). After 12 hours, donor animals were placed into the donor cage and sentinels (Sentinels 1, N = 8) were placed into the sentinel cage (1:1) at a 16.5 cm distance with an airflow of 30 cage changes/h as described by Port et al. [23]. Hamsters were co-housed for 24 h. The following day, donor animals were re-housed into regular rodent caging. One day later, Sentinels 1 were placed into the donor cage of new transmission set-ups. New sentinels (Sentinels 2, N = 8) were placed into the sentinel cage (2:2) at a 16.5 cm distance with an airflow of 30 changes/h. Hamsters were co-housed for 24 h. Then, Sentinels 1 were re-housed into regular rodent caging and Sentinels 2 were placed into the donor cage of new transmission set-ups one day later. New sentinels (Sentinels 3, N = 8) were placed into the sentinel cage (2:2) at a 16.5 cm distance with an airflow of 30 changes/h. Hamsters were co-housed for 24 h. Then both Sentinels 2 and Sentinels 3 were re-housed to regular rodent caging and monitored until 5 DPE. Oropharyngeal swabs were taken for all animals at 2 and 5 DPI/DPE. All animals were euthanized at 5 DPI/DPE for collection of lung tissue and nasal turbinates. To ensure no cross-contamination, the donor cages and the sentinel cages were never opened at the same time, sentinel hamsters were not exposed to the same handling equipment as donors, and the equipment was disinfected with either 70% EtOH or 5% Microchem after each sentinel. Regular bedding was replaced by alpha-dri bedding to avoid the generation of dust particles.

### Viral RNA detection

Swabs from hamsters were collected as described above. 140 μL was utilized for RNA extraction using the QIAamp Viral RNA Kit (Qiagen) using QIAcube HT automated system (Qiagen) according to the manufacturer’s instructions with an elution volume of 150 μL. For tissues, RNA was isolated using the RNeasy Mini kit (Qiagen) according to the manufacturer’s instructions and eluted in 60 μL. Sub-genomic (sg) and genomic (g) viral RNA were detected by qRT-PCR [54, 55]. RNA was tested with TaqMan^™^ Fast Virus One-Step Master Mix (Applied Biosystems) using QuantStudio 6 or 3 Flex Real-Time PCR System (Applied Biosystems). SARS-CoV-2 standards with known copy numbers were used to construct a standard curve and calculate copy numbers/mL or copy numbers/g.

### Viral titration

Viable virus in tissue samples was determined as previously described [15]. In brief, lung tissue samples were weighed, then homogenized in 1 mL of DMEM (2% FBS). Swabs were used undiluted. VeroE6 cells were inoculated with ten-fold serial dilutions of homogenate, incubated for 1 hour at 37°C, and the first two dilutions washed twice with 2% DMEM. For swab samples, cells were inoculated with ten-fold serial dilutions and no wash was performed. After 6 days, cells were scored for cytopathic effect. TCID_50_/mL was calculated by the Spearman-Karber method. To determine titers in air samples, a plaque assay was used. VeroE6 cells were inoculated with 200 uL/well (48-well plate) of undiluted samples, no wash was performed. Plates were spun for 1 h at RT at 1000 rpm. 800 ul of CMC (500 mL MEM (Cat#10370, Gibco, must contain NEAA), 5 mL PenStrep, 7.5 g carboxymethylcellulose (CMC, Cat# C4888, Sigma, sterilize in autoclave) overlay medium was added to each well and plates incubated for 6-days at 37°C. Plates were fixed with 10% formalin overnight, then rinsed and stained with 1% crystal violet for 10 min. Plaques were counted.

### Within-host kinetics model

We used Bayesian inference to fit a semi-mechanistic model of within-host virus kinetics and shedding to our data from inoculated hamsters. Briefly, the model assumes a period of exponential growth of virus within the host up to a peak viral load, followed by exponential decay. It assumes virus shedding into the air follows similar dynamics, and the time of peak air shedding and peak swab viral load may be offset from each other by an inferred factor. Decay of RNA may be slower than that of infectious virus by an inferred factor, representing the possibility, seen in our data, that some amplified RNA may be residual rather than representative of current infectious virus levels. We also inferred conversion factors (ratios) among the various quantities, i.e., how many oral swab sgRNA copies correspond to an infectious virion at peak viral load. We fit the model to our swab and cage air sample data using Numpyro [56], which implements a No-U-Turn Sampler [57]. For full mathematical details of the model and how it was fit, including prior distribution choices and predictive checks, see the Supplementary Appendix Methods.

### Serology

Serum samples were analyzed as previously described [58]. In brief, maxisorp plates (Nunc) were coated with 50 ng spike protein (generated in-house) per well. Plates were incubated overnight at 4°C. Plates were blocked with casein in phosphate buffered saline (PBS) (ThermoFisher) for 1 hours at room temperature (RT). Serum was diluted 2-fold in blocking buffer and samples (duplicate) were incubated for 1 hour at RT. Secondary goat anti-hamster IgG Fc (horseradish peroxidase (HRP)-conjugated, Abcam) spike-specific antibodies were used for detection and KPL TMB 2-component peroxidase substrate kit (SeraCare, 5120–0047) was used for visualization. The reaction was stopped with KPL stop solution (Seracare) and plates were read at 450 nm. The threshold for positivity was calculated as the average plus 3 × the standard deviation of negative control hamster sera.

### MesoPlex Assay

The V-PLEX SARS-CoV-2 Panel 13 (IgG) kit from Meso Scale Discovery was used to test binding antibodies against spike protein of SARS-CoV-2 with serum obtained from hamsters 14 DPI diluted at 10,000X. A standard curve of pooled hamster sera positive for SARS-CoV-2 spike protein was serially diluted 4-fold. The secondary antibody was prepared by conjugating a goat anti-hamster IgG cross-adsorbed secondary antibody (ThermoFisher) using the MSD GOLD SULFO-TAG NHS-Ester Conjugation Pack (MSD). The secondary antibody was diluted 10,000X for use on the assay. The plates were prepped, and samples were run according to the kit’s instruction manual. After plates were read by the MSD instrument, data was analyzed with the MSD Discovery Workbench Application.

### Virus neutralization

Heat-inactivated γ-irradiated sera were two-fold serially diluted in DMEM. 100 TCID_50_ of SARS-CoV-2 strain England/204820464/2020 (B.1.1.7/Alpha, EPI_ISL_683466) or hCoV-19/USA/KY-CDC-2–4242084/2021 (B.1.617.2/Delta, EPI_ISL_1823618) was added. After 1 h of incubation at 37°C and 5% CO_2_, the virus:serum mixture was added to VeroE6 cells. CPE was scored after 5 days at 37°C and 5% CO_2_. The virus neutralization titer was expressed as the reciprocal value of the highest dilution of the serum that still inhibited virus replication. The antigenic map was constructed as previously described [59, 60] using the antigenic cartography software from https://acmacs-web.antigenic-cartography.org. In brief, this approach to antigenic mapping uses multidimensional scaling to position antigens (viruses) and sera in a map to represent their antigenic relationships. The maps here relied on the first SARS-CoV-2 infection serology data of Syrian hamsters. The positions of antigens and sera were optimized in the map to minimize the error between the target distances set by the observed pairwise virus-serum combinations. Maps were effectively constructed in only one dimension because sera were only titrated against two viruses and the dimensionality of the map is constrained to the number of test antigens minus one.

### Next-generation sequencing of virus

Total RNA was extracted from oral swabs, lungs, and nasal turbinates using the Qia Amp Viral kit (Qiagen, Germantown, MD), eluted in EB, and viral Ct values were calculated using real-time PCR. Subsequently, 11 μL of extracted RNA was used as a template in the ARTIC nCoV-2019 sequencing protocol V.1 (Protocols.io - https://www.protocols.io/view/ncov-2019-sequencing-protocol-bbmuik6w) to generate 1st-strand cDNA. Five microliters were used as template for Q5 HotStart Pol PCR (Thermo Fisher Sci, Waltham, MA) together with 10 uM stock of a single primer pair from the ARTIC nCoV-2019 v3 Panel (Integrated DNA Technologies, Belgium), specifically 76L_alt3 and 76R_alt0. Following 35 cycles and 55°C annealing temperature, products were AmPure XP cleaned and quantitated with Qubit (Thermo Fisher Sci) fluorometric quantitation per instructions. Following visual assessment of 1 ul on a Tape Station D1000 (Agilent Technologies, Santa Clara, CA), a total of 400 ng of product was taken directly into TruSeq DNA PCR-Free Library Preparation Guide, Revision D (Illumina, San Diego, CA) beginning with the Repair Ends step (q.s. to 60 μL with RSB). Subsequent cleanup consisted of a single 1:1 AmPure XP/reaction ratio and all steps followed the manufacturer’s instructions including the Illumina TruSeq CD (96) Indexes. Final libraries were visualized on a BioAnalyzer HS chip (Agilent Technologies) and quantified using KAPA Library Quant Kit - Illumina Universal qPCR Mix (Kapa Biosystems, Wilmington, MA) on a CFX96 Real-Time System (BioRad, Hercules, CA). Libraries were diluted to 2 nM stock, pooled together in equimolar concentrations, and sequenced on the Illumina MiSeq instrument (Illumina) as paired-end 2 × 250 base pair reads. Because of the limited diversity of a single-amplicon library, 20% PhiX was added to the final sequencing pool to aid in final sequence quality. Raw fastq reads were trimmed of Illumina adapter sequences using cutadapt version 1.1227, then trimmed and filtered for quality using the FASTX-Toolkit (Hannon Lab, CSHL). To process the ARTIC data, a custom pipeline was developed [61]. Fastq read pairs were first compared to a database of ARTIC primer pairs to identify read pairs that had correct, matching primers on each end. Once identified, the ARTIC primer sequence was trimmed off. Read pairs that did not have the correct ARTIC primer pairs were discarded. Remaining read pairs were collapsed into one sequence using AdapterRemoval [62] requiring a minimum 25 base overlap and 300 base minimum length, generating ARTIC amplicon sequences. Identical amplicon sequences were removed, and the unique amplicon sequences were then mapped to the SARS-CoV-2 genome (MN985325.1) using Bowtie2 [63]. Aligned SAM files were converted to BAM format, then sorted and indexed using SAMtools [64]. Variant calling was performed using Genome Analysis Toolkit (GATK, version 4.1.2) HaplotypeCaller with ploidy set to 2 [65]. Single nucleotide polymorphic variants were filtered for QUAL > 200 and quality by depth (QD) > 20 and indels were filtered for QUAL > 500 and QD > 20 using the filter tool in bcftools, v1.9 [64].

### Histopathology

Necropsies and tissue sampling were performed according to IBC-approved protocols. Tissues were fixed for a minimum of 7 days in 10% neutral buffered formalin with 2 changes. Tissues were placed in cassettes and processed with a Sakura VIP-6 Tissue Tek on a 12-hour automated schedule using a graded series of ethanol, xylene, and ParaPlast Extra. Prior to staining, embedded tissues were sectioned at 5 μm and dried overnight at 42°C. Using GenScript U864YFA140–4/CB2093 NP-1 (1:1000), specific anti-CoV immunoreactivity was detected using the Vector Laboratories ImPress VR anti-rabbit IgG polymer (# MP-6401) as secondary antibody. The tissues were then processed using the Discovery Ultra automated processor (Ventana Medical Systems) with a ChromoMap DAB kit Roche Tissue Diagnostics (#760–159).

### Statistical Analysis

Significance tests were performed as indicated where appropriate for the data. Unless stated otherwise, statistical significance levels were determined as follows: ns = p > 0.05; * = p ≤ 0.05; ** = p ≤ 0.01; *** = p ≤ 0.001; **** = p ≤ 0.0001. Exact nature of tests is stated where appropriate.

### Data availability statement

Data to be deposited in Figshare.

## Supplementary Material

Supplement 1

## Significance

Airborne transmission is one of the major routes for SARS-CoV-2, however underlying host and virus parameters remain poorly understood. Here, we provide direct experimental evidence that the quantitative and qualitative assessment of exhaled aerosols are critical to understand the efficiency of SARS-CoV-2 airborne transmission. We show that after infection, the Alpha and Delta variants of concern displayed a short window of detectable virus in the air in contrast to prolonged shedding measured in oropharyngeal swabs. The limited window coincided with changes in airway constriction, and a sex dependent decrease of fine aerosols produced in the 1–10 μm aerodynamic diameter range. Dual airborne infections only occurred when both viruses were shed by the same donor and after prolonged exposure.
