# Supplementary material for: Host and viral determinants of airborne transmission of SARS-CoV-2 in the Syrian hamster
Source: bioRxiv. 2022 Aug 15:2022.08.15.504010. Preprint. [Version 1] doi: 10.1101/2022.08.15.504010 (PMC9413705; doi:10.1101/2022.08.15.504010)
Supplement: Supplementary file 1 [file media-1.pdf]

Supporting Information for

Host and viral determinants of airborne transmission of SARS-CoV-2 in the Syrian hamster.

Julia R. Port*[1], Dylan H. Morris[2], Jade C. Riopelle[1], Claude Kwe Yinda[1], Victoria A. Avanzato[1], Myndi G. Holbrook[1], Trenton Bushmaker[1], Jonathan E. Schulz[1], Taylor A. Saturday[1], Kent Barbian[3], Colin A. Russell[4], Rose Perry-Gottschalk[5], Carl I. Shaia[6], Craig Martens[3], James O. Lloyd-Smith[2], Robert J. Fischer[1], Vincent J. Munster#[1]

1. *Laboratory of Virology, Division of Intramural Research, National Institute of Allergy and Infectious Diseases, National Institutes of Health, Hamilton, MT, USA*
2. *Department of Ecology and Evolutionary Biology, University of California, Los Angeles, CA, USA*
3. *Rocky Mountain Research and Technologies Branch, Division of Intramural Research, National Institute of Allergy and Infectious Diseases, National Institutes of Health, Hamilton, MT, USA*
4. *Department of Medical Microbiology | Amsterdam University Medical Center, University of Amsterdam*
5. *Rocky Mountain Visual and Medical Arts Unit, Research Technologies Branch, Division of Intramural Research, National Institute of Allergy and Infectious Diseases, National Institutes of Health, Hamilton, MT, USA*
6. *Rocky Mountain Veterinary Branch, Division of Intramural Research, National Institute of Allergy and Infectious Diseases, National Institutes of Health, Hamilton, MT, USA*

Corresponding author: Vincent J. Munster. vincent.munster@nih.gov

This PDF file includes:

Figures S1 to S7
Tables S1
SI References

Other supporting materials for this manuscript include the following:
SI Mathematical Model Methods

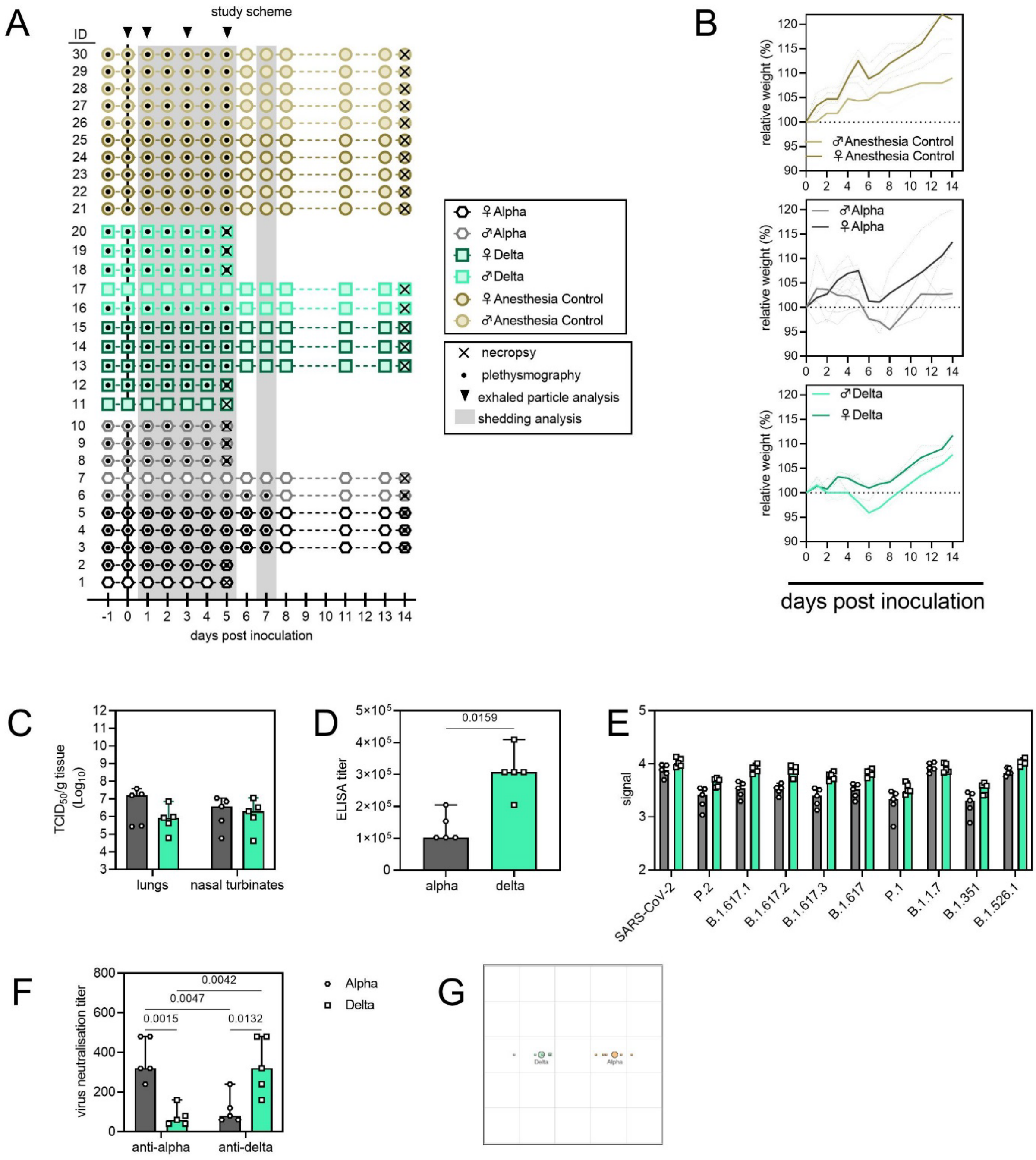

Figure S1. Schematic overview of inoculation experiment. A. Four-to-six-week-old female and male Syrian hamsters (ENVIGO) were inoculated (N = 10 per virus, per sex) with 10^3 $TCID_{50}$ intranasally (IN) with either SARS-CoV-2 Alpha or Delta variants, or no virus (anaesthesia controls). At five days post inoculation, five hamsters for each group were euthanized, and tissues were collected. The remaining 5 animals for each route were euthanized at 14 DPI for disease course assessment and shedding analysis. For the control group no day 5 necropsy was performed. Schematic indicates when oropharyngeal swabs were collected, when whole body plethysmography was performed, when air sampling was conducted and when exhaled particle profiles were determined for everyone. **B**. Relative weight loss. Graph shows median (thick line) and individuals, colors indicate sex, N = 10. **C**. Viral load as measured by infectious titers in lungs

and nasal turbinates collected at day 5 post inoculation. Bar-chart depicting median, 96% CI and individuals, N = 5, ordinary two-way ANOVA, followed by Šídák's multiple comparisons test. Dotted line = limit of detection. **D**. Binding antibodies against spike protein of SARS-CoV-2 in serum obtained 14 days post inoculation. Bar-chart depicting median, 96% CI and individuals, N = 5, Mann-Whitney test. ELISA was performed once. **E.** Binding antibodies against spike protein of various variants of concern analyzed by MesoPlex. Bar-chart depicting median, 96% CI and individuals, N = 5 ordinary two-way ANOVA, followed by Šídák's multiple comparisons test. Assay was performed once. **F.** Virus neutralization titers against Alpha and Delta, depicted as reciprocal titers. N = 5, ordinary two-way ANOVA, followed by Tukey's multiple comparisons test. Assay was performed once. = Alpha, teal = Delta, beige = anesthesia control. **G.** Antigenic map [1] depicting the cross-reactivity based on neutralization. grey p-values are indicated were significant. Abbreviations: g, genomic; sg, subgenomic; RBD, receptor binding domain; ACE2, Angiotensin-converting enzyme 2; ELISA, Enzyme-linked immune-absorbent Assay.

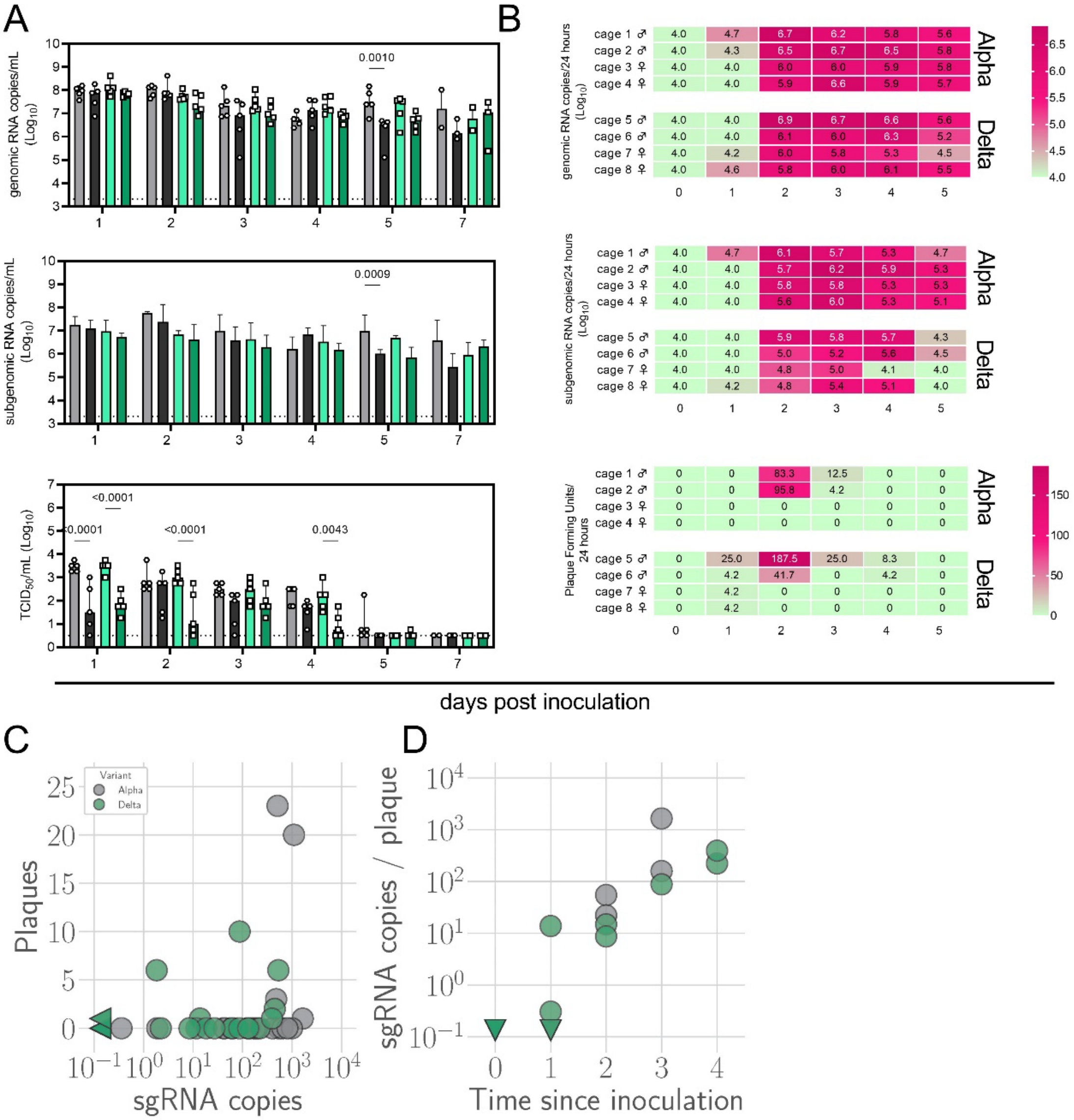

Figure S2. Window of Alpha and Delta variant shedding profiles. Syrian hamsters were inoculated with 10^3 $TCID_{50}$ via the intranasal route with Alpha or Delta. **A.** Viral load as measured by gRNA, sgRNA and infectious titers in oropharyngeal swabs collected at 1, 2 ,3 ,4-, 5- and 7-days post inoculation. Bar-chart depicting median, 96% CI and individuals, N = 5, ordinary two-way ANOVA, followed by Šídák's multiple comparisons test. Dotted line = limit of detection. **B.** Virus isolated from cage air over 24 h intervals, measured as gRNA, sgRNA and plaque forming units on day 0, 1, 2, 3, 4, and 5. The column marked 1 corresponds to samples taken from 0-24 hours post inoculation. Each cage housed 2 or 3 hamsters. Heatmap depicting individual cages across each day, colours referring to legends on the right. RNA: limit of detection = 4.0, Plaque forming units: limit of detection = 0. **C.** sgRNA sampled from air versus infectious virus sampled from air. Point colour indicates variant: Alpha (grey) or Delta (green). Sampled sgRNA copies value versus sampled plaques. **D.** Number of estimated sgRNA copies per plaque in samples as a function of day sampled and variant. Grey = Alpha, teal = Delta, dark = female, light = males. p-values are indicated where significant. Abbreviations: g, genomic; sg, subgenomic; TCID, Tissue Culture Infectious Dose.

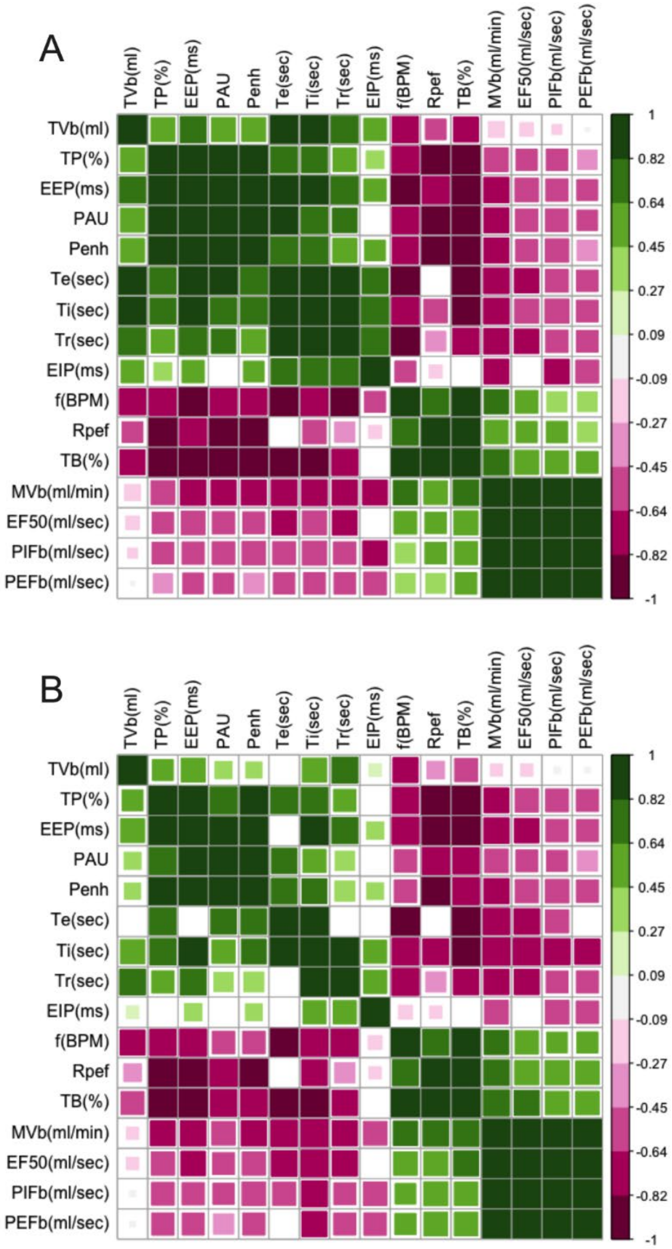

Figure S3. Lung function changes after SARS-CoV-2 inoculation. Lung function was assessed on day -1, 0, 1, 2, 3, 4 and 5 by whole body plethysmography. **A, B.** Correlation plots depicting correlations of relevant parameters for Alpha (A) and Delta (B) inoculated groups. Abbreviations: TVb, tidal volume; TP, duration of pause before inspiration as a percentage of total breath duration; EEP, end expiratory pause; PAU, index of constriction; Penh, index of constriction; Te, expiratory time; Ti, inspiratory time; Tr, relaxation time; EIP, end inspiratory pause; f, frequency; Rpef, time to reach peak expiratory flow/total expiration time; TB, duration of pause before expiration as a percentage of total breath duration; MVb, minute volume; EF50, expiratory flow at 50% expired volume; PIFb, peak inspiratory flow; PEFb, peak expiratory flow.

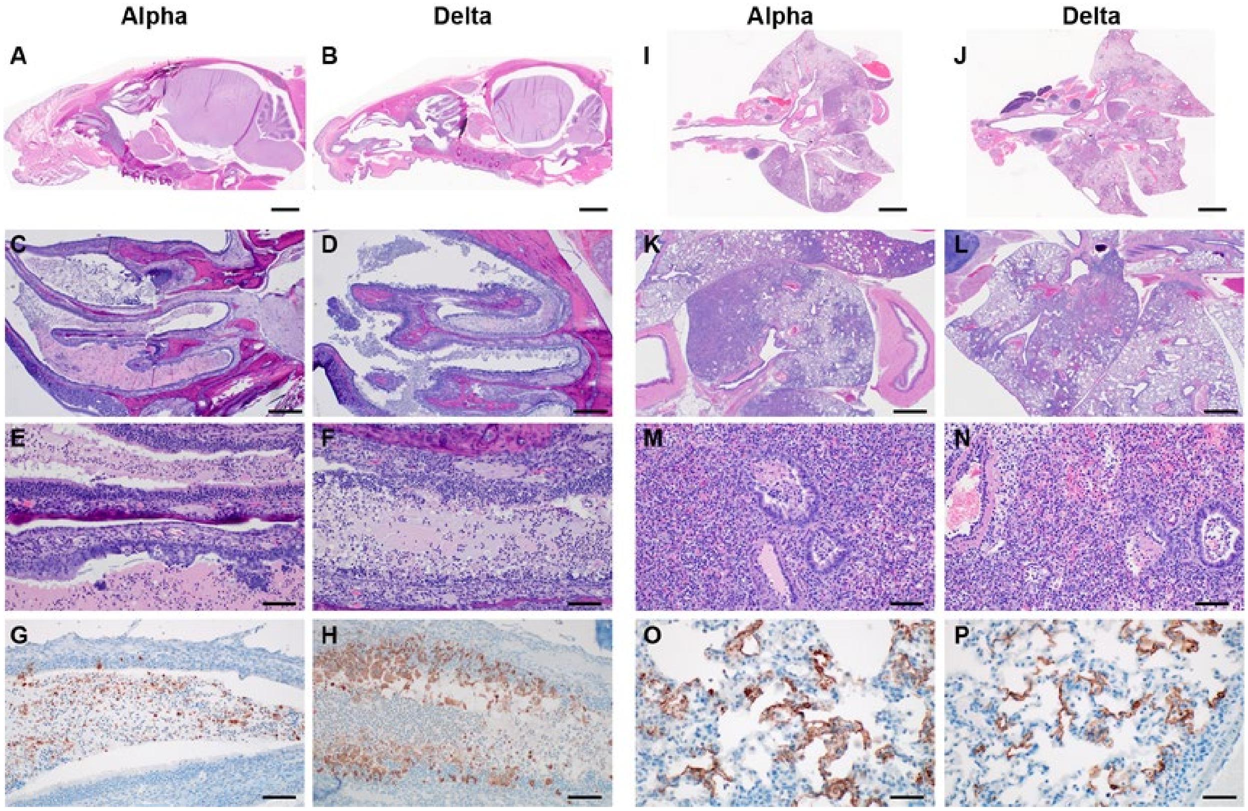

Figure S4. Respiratory tract pathology after SARS-CoV-2 infection with Alpha and Delta. Syrian hamsters were inoculated with 10^2 $TCID_{50}$ via the intranasal route with Alpha or Delta and respiratory pathology compared on day 5. **A, B.** Skull, brain, nasal turbinates and cavities, Alpha and Delta variant, 1x HE. **C, D**. Nasal cavities contain an exudate and are lined by inflamed, disrupted and necrotic epithelium, 40x HE. **E, F**. Eroded and ulcerated olfactory epithelium, epithelial micro-abscesses, sloughed epithelial and inflammatory exudate occupies nasal cavities and recesses (*), 200x HE. **G, H**. Examples of immunoreactivity in the exudate (*) of the nasal cavity from an Alpha and Delta hamster. There are also rare immunoreactive olfactory epithelial cells in the Alpha variant sample and numerous immunoreactive epithelial cells in the Delta variant sample. 200x, anti SARS-2 IHC. **I, J.** Lung, Score 4, Alpha and Delta variant, 1x HE. **K, L.** Foci of inflammation across multiple lobes, 20x HE. **M, N**. Foci of inflammation contain numerous inflammatory cells as well as hemorrhage, fibrin and edema. Bronchioles contain fibrin, sloughed epithelial and inflammatory cells (*). Vessels contain sub-intimal leukocytes and vessel walls are occasionally infiltrated by leukocytes (arrows), 200x HE. O, P. Alveolar immunoreactivity from an Alpha and Delta hamster. 400x, anti SARS-2 IHC. Mainly, severe inflammation and not yet well-developed interstitial pneumonia was observed. Foci were consolidated but only rarely contained hyperplastic type II pneumocytes and syncytial cells. The large and mid-caliber bronchioles were frequently lined by hyperplastic respiratory epithelium mixed with rare singular necrotic cells and transmigrating leukocytes. Lesions were associated with hemorrhage, fibrin and edema. Vessels also contained sub-endothelial clusters of leukocytes within the muscular layers and surrounding adventitia. In the nasal turbinates SARS-CoV-2 immunoreactivity was seen in the exudate of the nasal and rarely in olfactory epithelium, regardless of VOC. No sex-specific increases in pathology were observed.

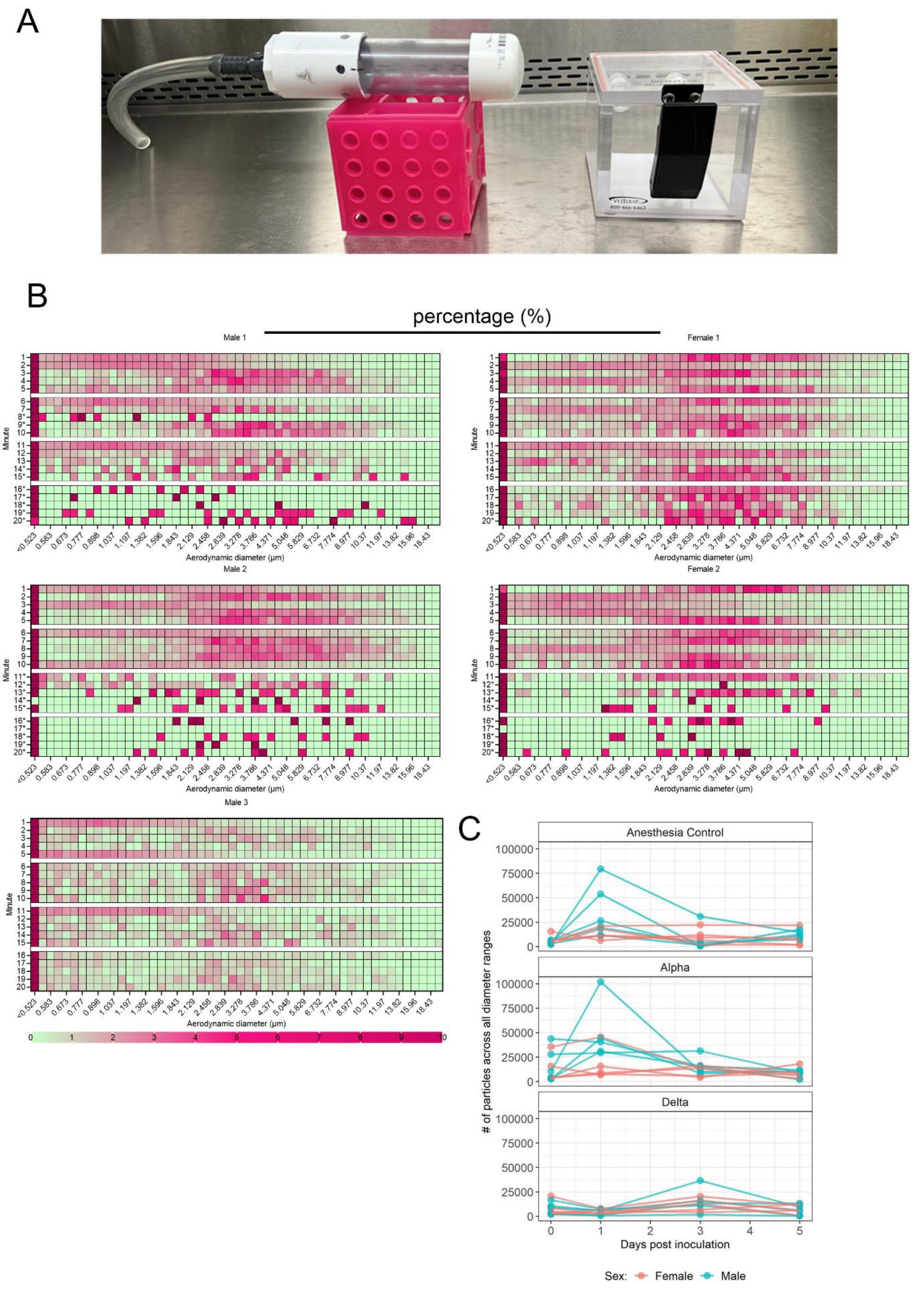
A
B
percentage (%)
Male 1
Female 1
Male 2
Female 2
Male 3
Minute
Aerodynamic diameter (µm)
C
Anesthesia Control
Alpha
Delta
# of particles across all diameter ranges
Days post inoculation
Sex: Female Male

Figure S5. Exhaled particle profiles of Syrian hamsters A. Chambers used to house uninfected hamsters (left) and animals during inoculation experiments (inoculation with Alpha, Delta or control inoculum) (right). **B.** Uninfected healthy animals were used to assess particle profiles in relation to behavior patterns. Five Syrian hamsters were acclimatized to a small tube, in which animal movement was limited and air flow was directly passing the face. For each animal, 5 x 5-minute readings were taken. Heatmap shows the percentage of total particles in each diameter size for 3 male and 2 female animals. * marks minutes of low to no activity (sleep). Colors refer to scale below. **C**. Syrian hamsters were inoculated with 10^2 $TCID_{50}$ via the intranasal route with Alpha (N = 10) or Delta (N = 10). Aerodynamic diameter profile of exhaled particles was analyzed on day 0, 1, 3, and 5. For each animal (N = 10 in each variant group, comprising 5 makes and 5 females), line graph of the total number of particles by variant and sex indicated by color (red=female; blue=male).

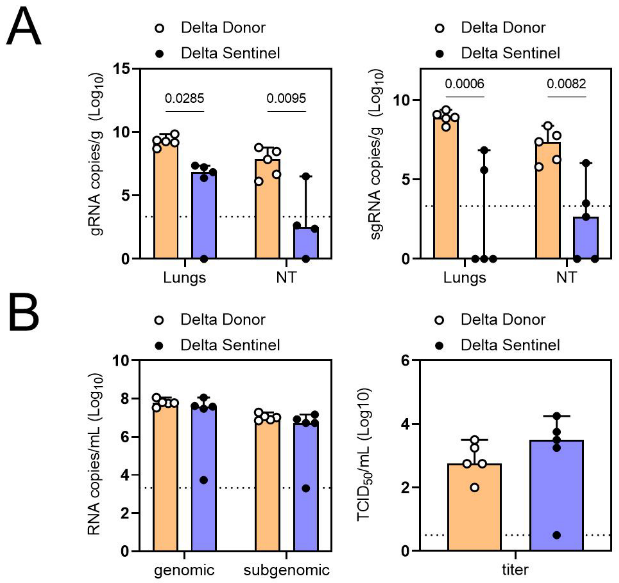

Figure S6. Early virus shedding in donors and sentinels. Donor animals (N = 10) were inoculated with Delta variant with 10^2 $TCID_{50}$ via the intranasal route. Sentinels (1:1 ratio) were exposed subsequently at 16.5 cm distance for 24 h, beginning 24 h after donor exposure. **A.** Organ titers measured by gRNA and sgRNA on day 2 post inoculation/exposure. **B.** Respiratory shedding measured by viral load in oropharyngeal swabs; measured by gRNA, sgRNA and infectious titers on day 2 post inoculation/exposure. Bar-chart depicting median, 96% CI and individuals, N = 5, ordinary two-way ANOVA, followed by Šídák's multiple comparisons test and Wilcoxon test. Orange = donors, purple = sentinels, p-value shown where significant.

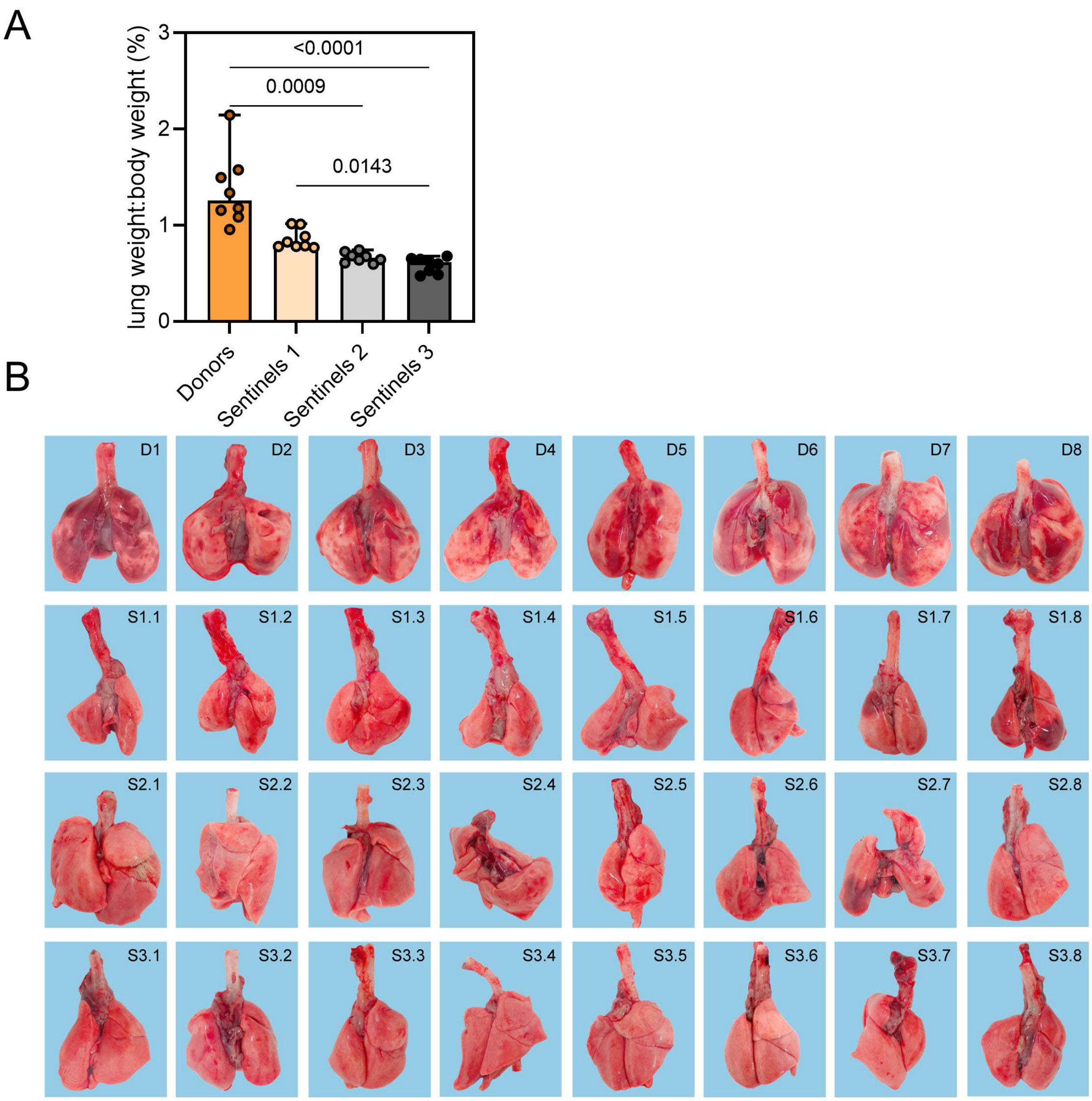

Figure S7. Lung pathology reduction across airborne transmission chain. Donor animals (N = 8) were inoculated with Alpha and Delta variant with 10^2 $TCID_{50}$ via the intranasal route (1:1 ratio), and three groups of sentinels (Sentinels 1, 2 and 3) were exposed subsequently at 16.5 cm distance. Animals were exposed at a 1:1 ratio, exposure occurred on day 1 (Donors → Sentinels 1) and day 2 (Sentinels → Sentinels) and lasted for 24 hours for each chain link. A. Severity of lung pathology on day 5 measured in lung:body weight . Bar-chart depicting median, 96% CI and individuals, N = 8, ordinary two-way ANOVA, followed by Šídák's multiple comparisons test. Dark orange = Donors, light orange = Sentinels 1, grey = Sentinels 2, dark grey = Sentinels 3, p-values are indicated were significant. **B.** Gross image of individual lungs. Donor lungs failed to collapse completely and have mottled, hemorrhagic foci on their surface. Sentinel lungs have fewer lesions and less mottling and hemorrhage.

Table S1: Airborne competitiveness of Alpha and Delta SARS-CoV-2 variants. Donor animals (N = 8) were inoculated with Alpha and Delta variant with 10^2 $TCID_{50}$ via the intranasal route (1:1 ratio), and three groups of sentinels (Sentinels 1, 2 and 3) were exposed subsequently at 16.5 cm distance. Animals were exposed at a 1:1 ratio, exposure occurred on day 1 (Donors → Sentinels 1) and day 2 (Sentinels → Sentinels) and lasted for 24 hours for each chain link. Percentage of reads mapped to Delta for swabs and tissue samples collected for each animal. Percentage represents average between 3 SNPs.

Tissue	Subject	Percentage Delta
swab 2 DPI/DPE	Donor 1	56.33333
swab 2 DPI/DPE	Donor 2	47.33333
swab 2 DPI/DPE	Donor 3	40.33333
swab 2 DPI/DPE	Donor 4	69
swab 2 DPI/DPE	Donor 5	56.66667
swab 2 DPI/DPE	Donor 6	45
swab 2 DPI/DPE	Donor 7	62
swab 2 DPI/DPE	Donor 8	59.66667
swab 2 DPI/DPE	Sentinel 1.1	92.66667
swab 2 DPI/DPE	Sentinel 1.2	92.33333
swab 2 DPI/DPE	Sentinel 1.3	19
swab 2 DPI/DPE	Sentinel 1.4	92.33333
swab 2 DPI/DPE	Sentinel 1.5	63.66667
swab 2 DPI/DPE	Sentinel 1.6	80.3
swab 2 DPI/DPE	Sentinel 1.7	89.66667
swab 2 DPI/DPE	Sentinel 1.8	85
swab 2 DPI/DPE	Sentinel 2.1	NA
swab 2 DPI/DPE	Sentinel 2.2	16
swab 2 DPI/DPE	Sentinel 2.3	NA
swab 2 DPI/DPE	Sentinel 2.4	NA
swab 2 DPI/DPE	Sentinel 2.5	12
swab 2 DPI/DPE	Sentinel 2.6	NA
swab 2 DPI/DPE	Sentinel 2.7	91.66667
swab 2 DPI/DPE	Sentinel 2.8	92
swab 5 DPI/DPE	Donor 1	41.66667
swab 5 DPI/DPE	Donor 2	34.33333
swab 5 DPI/DPE	Donor 3	67.66667
swab 5 DPI/DPE	Donor 4	36
swab 5 DPI/DPE	Donor 5	NA
swab 5 DPI/DPE	Donor 6	65
swab 5 DPI/DPE	Donor 7	61.66667
swab 5 DPI/DPE	Donor 8	60
swab 5 DPI/DPE	Sentinel 1.1	92
swab 5 DPI/DPE	Sentinel 1.2	20.5
swab 5 DPI/DPE	Sentinel 1.3	17
swab 5 DPI/DPE	Sentinel 1.4	91.33333
swab 5 DPI/DPE	Sentinel 1.5	20.33333
swab 5 DPI/DPE	Sentinel 1.6	92.33333

swab 5 DPI/DPE	Sentinel 1.7	91.33333
swab 5 DPI/DPE	Sentinel 1.8	45.33333
swab 5 DPI/DPE	Sentinel 2.1	92
swab 5 DPI/DPE	Sentinel 2.2	17
swab 5 DPI/DPE	Sentinel 2.3	18.5
swab 5 DPI/DPE	Sentinel 2.4	NA
swab 5 DPI/DPE	Sentinel 2.5	18.5
swab 5 DPI/DPE	Sentinel 2.6	NA
swab 5 DPI/DPE	Sentinel 2.7	91.66667
swab 5 DPI/DPE	Sentinel 2.8	92.66667
swab 5 DPI/DPE	Sentinel 3.1	NA
swab 5 DPI/DPE	Sentinel 3.2	NA
swab 5 DPI/DPE	Sentinel 3.3	NA
swab 5 DPI/DPE	Sentinel 3.4	NA
swab 5 DPI/DPE	Sentinel 3.5	18
swab 5 DPI/DPE	Sentinel 3.6	NA
swab 5 DPI/DPE	Sentinel 3.7	NA
swab 5 DPI/DPE	Sentinel 3.8	NA
nasal turbinates	Donor 1	52.66667
nasal turbinates	Donor 2	38.66667
nasal turbinates	Donor 3	59
nasal turbinates	Donor 4	89.33333
nasal turbinates	Donor 5	48.33333
nasal turbinates	Donor 6	49.66667
nasal turbinates	Donor 7	67
nasal turbinates	Donor 8	42
nasal turbinates	Sentinel 1.1	94.66667
nasal turbinates	Sentinel 1.2	13.66667
nasal turbinates	Sentinel 1.3	0
nasal turbinates	Sentinel 1.4	91.33333
nasal turbinates	Sentinel 1.5	0
nasal turbinates	Sentinel 1.6	94
nasal turbinates	Sentinel 1.7	95
nasal turbinates	Sentinel 1.8	91.66667
nasal turbinates	Sentinel 2.1	55
nasal turbinates	Sentinel 2.2	NA
nasal turbinates	Sentinel 2.3	12.5
nasal turbinates	Sentinel 2.4	11.5
nasal turbinates	Sentinel 2.5	11
nasal turbinates	Sentinel 2.6	12
nasal turbinates	Sentinel 2.7	55.66667
nasal turbinates	Sentinel 2.8	95.33333
lung	Donor 1	39.33333
lung	Donor 2	55.33333

lung	Donor 3	44.66667
lung	Donor 4	75.66667
lung	Donor 5	25.33333
lung	Donor 6	23.33333
lung	Donor 7	40
lung	Donor 8	74.33333
lung	Sentinel 1.1	94.66667
lung	Sentinel 1.2	12
lung	Sentinel 1.3	13
lung	Sentinel 1.4	94.66667
lung	Sentinel 1.5	19.33333
lung	Sentinel 1.6	95
lung	Sentinel 1.7	95.33333
lung	Sentinel 1.8	73.66667
lung	Sentinel 2.1	NA
lung	Sentinel 2.2	NA
lung	Sentinel 2.3	12
lung	Sentinel 2.4	14.33333
lung	Sentinel 2.5	11
lung	Sentinel 2.6	NA
lung	Sentinel 2.7	95.33333
lung	Sentinel 2.8	NA

SI References

1. Smith, D.J., et al., *Mapping the Antigenic and Genetic Evolution of Influenza Virus.* Science, 2004. **305**(5682): p. 371-376.

Supplementary text: within-host dynamics model and Bayesian infererence methods

Contents

1 Introduction **2**
1.1 Notation . . . 2
1.2 Units . . . 3

2 Dynamics model **3**
2.1 Growth and decay of air shedding . . . 3
2.2 Offset growth and decay of oral shedding . . . 3
2.3 Relationship between sgRNA and infectious virus . . . 4
2.4 Initial shedding value . . . 4
2.5 Variant effects . . . 5
2.6 Respiration rates . . . 5
2.7 Sex effects . . . 5
2.8 Individual heterogeneity in disease course . . . 5

3 Predicting observable quantities **6**
3.1 Units of virus dynamics . . . 6
3.2 Converting predicted swab virus to units of TCID . . . 6
3.3 Predicting air sample plaques . . . 7
3.4 Predicting air sample sgRNA . . . 8
3.5 Predicting sentinel exposures . . . 9

4 Relating predicted quantities to observed quantities **9**
4.1 Oral swabs . . . 9
4.2 Air samples . . . 10
4.3 Respiration rates . . . 11
4.4 Sentinel infection status . . . 11

5 Prior distributions **11**
5.1 Respiration rates . . . 12
5.2 Virological parameters . . . 12
5.3 Sentinel infection process constant . . . 13
5.4 Observation error standard deviations . . . 13

6 **Assessing coinfection probabilities** 13
6.1 Results 14

7 **Computational methods** 17

8 **Additional mathematical details** 17
8.1 Well observation process 17

1 Introduction

We modeled the observation process explicitly to extract maximal information from the data generated.

1.1 Notation

In the text that follows, we use the following mathematical notation.

1.1.1 Logarithms and exponentials

$\log(x)$ denotes the logarithm base e of x (sometimes called $\ln(x)$). We explicitly refer to the logarithm base 10 of x as $\log_{10}(x)$. $\exp(x)$ denotes e^x.

1.1.2 Probability distributions

The symbol $\sim$ denotes that a random variable is distributed according to a given probability distribution. So for example

$$X \sim \text{Normal}(0, 1)$$

indicates that the random variable X is normally distributed with mean 0 and standard deviation 1.

We parameterize normal distributions as:

$$\text{Normal}(\text{mean}, \text{standard deviation})$$

We parameterize positive- and negative-constrained normal distributions (i.e. with lower limit 0 and upper limit zero, respectively) as:

$$\text{PosNormal}(\text{mode}, \text{standard deviation})$$
$$\text{NegNormal}(\text{mode}, \text{standard deviation})$$

We parameterize censored normal distributions, in which values outside the censoring range are reported at the lower limit of detection (lld) and upper limit of detection (uld), respectively, as:

$$\text{Censored Normal}(\text{mean}, \text{standard deviation}, \text{lld}, \text{uld})$$

We parameterize Poisson distributions as:

$$\text{Poisson}(\text{mean})$$

1.2 Units

Unless otherwise stated, we express time in units of hours, volume in units of mL, infectious virus in units of plaque forming units collected on our air filter, and sgRNA in units of copy numbers.

2 Dynamics model

We modeled the within-host dynamics of the virus within inoculated hamsters as a process of exponential growth of virus up to a peak, followed by exponential decay of virus down from that peak. The principal quantity of interest is airborne virus shedding over time $V_a(t)$, expressed in units of infectious virions exhaled per unit volume of exhaled air per unit time. We express time in units of hours post-infection.

2.1 Growth and decay of air shedding

We denote the exponential growth rate of the virus within the hamster by g and the exponential decay rate after the peak by d_{av}. We denote the time of peak airborne shedding $t_a > 0$ and define $t = 0$ as the time of inoculation.

Our model is therefore:

$$V_a(t) = \begin{cases} V_a(0) \exp\left[gt\right] & t < t_a \\ V_a(0) \exp\left[gt_p - d_{av}(t - t_a)\right] & t \geq t_a \end{cases} \tag{1}$$

2.2 Offset growth and decay of oral shedding

Since we also took measurements of virus shed in oral swabs, we incorporated the dynamics of oral swab shedding $V_o(t)$ into our model. We modeled $V_o(t)$ as offset in time from the dynamics of airborne shedding $V_a(t)$ by some offset factor $\omega > 0$. That is, the time of peak oral shedding t_o is:

$$t_o = \omega t_a \tag{2}$$

Note that $\omega < 1$ implies that swab shedding peaks earlier than airborne shedding, $\omega > 1$ implies that swab shedding peaks later, and $\omega = 0$ implies the peaks coincide in time.

We also allowed for the possibility that oral virus shedding decays at a faster or slower rate d_{ov} than airborne virus shedding, which decays at a rate d_{av}. Specifically, we defined the ratio of d_{ov} to d_{av} as $q_o > 0$, so:

$$d_{ov} = q_o d_{av} \tag{3}$$

Then:

$$V_o(t) = \begin{cases} V_o(0) \exp\left[gt\right] & t < t_o \\ V_o(0) \exp\left[gt_o - d_{ov}(t - t_o)\right] & t \geq t_o \end{cases} \tag{4}$$

2.3 Relationship between sgRNA and infectious virus

We also modeled the possibility that measured sgRNA decays slower or faster than measured infectious virus. Slower decay, for instance, could result from persistence of undegraded RNA after all infectious virions have been neutralized or otherwise lost infectivity.

We modeled this possibly different RNA shedding decay rate with an estimated ratio $q_n > 0$ that relates sgRNA shedding decay to infectious virus shedding shedding decay. So the decay rate of air sgRNA shedding d_{an} is:

$$d_{an} = q_n d_{av} \tag{5}$$

And similarly the decay rate of oral sgRNA shedding d_{on} is:

$$d_{on} = q_n d_{ov} = q_o q_n d_{av} \tag{6}$$

We modeled the ratio between produced virus and produced sgRNA copies with multipliers o_n for oral swabs and a_n for air samples. So before the decay phase begins, $V(t)$ and $N(t)$ are linearly related.

$$\begin{aligned} N_a(t) &= a_n V_a(t) \quad \text{if } t < t_a \\ N_o(t) &= o_n V_o(t) \quad \text{if } t < t_o \end{aligned} \tag{7}$$

The dynamics of airborne sgRNA shedding $N_a(t)$ and oral sgRNA shedding $N_o(t)$ are therefore equivalent to those for infectious virus in equations 1 and 4, respectively, but with d_{an} instead of d, d_{on} instead of d_{ov}, and initial values $N_a(0) = a_n V_a(0)$, $N_o(0) = o_n V_o(0)$:

$$N_a(t) = \begin{cases} N_a(0) \exp\left[gt\right] & t < t_a \\ N_a(0) \exp\left[gt_a - d_{an}(t - t_a)\right] & t \geq t_a \end{cases} \tag{8a}$$

$$N_o(t) = \begin{cases} N_o(0) \exp\left[gt\right] & t < t_o \\ N_o(0) \exp\left[gt_o - d_{on}(t - t_o)\right] & t \geq t_o \end{cases} \tag{8b}$$

2.4 Initial shedding value

For inference purposes, rather than set a prior distribution on the initial airborne shedding viral load $V_0 = V_a(0)$, we instead set a prior on the peak airborne viral load $V_{\max} = V_a(t_a)$ and back-calculated $V_a(0)$ (and thus $V_o(0)$, $N_a(0)$, and $N_o(0)$ via:

$$\log(V_0) = \log(V_{\max}) - gt_a \tag{9}$$

2.5 Variant effects

We allowed the two variants of interest, Alpha and Delta, to take on different typical values for all of the virological parameters: g, d_{av}, t_a, ω, q_o, q_n, a_n, o_n, and mean peak viral shedding $V_{\max}$. We denote the variant-specific values for variant i by g_i, d_{avi}, and so on.

2.6 Respiration rates

We also measured animal respiration rates $m(t)$ and included these in our model. The amount of infectious virus an animal deposits into the air per unit time is $m(t)\,V_a(t)$ and the amount of sgRNA the animal deposits per unit time is $m(t)N_a(t)$.

2.7 Sex effects

Since male hamsters are physically larger and appeared to have different shedding profiles, we wanted to be able to estimate the effect of host sex on key parameters of interest. To do this, we modeled males as possibly offset from females in their typical values of respiration rate $m(t)$, airborne shedding exponential growth rate g, airborne shedding exponential decay rate d_{av}, peak airborne shedding time t_a, and peak airborne shedding rate $V_{\max}$ (this then has downstream consequences for other parameters such as d_{an} or t_o that depend on those core virological parameters).

We modeled sex differences in the virological parameters via offsets to the mean log values for male hamsters. Δ_x denotes the offset for variable x. So for example if females have a mean log respiration rate of $\log[m]$, males have one of $\log[m] + \Delta_m$. We also estimated male offsets Δ_g for growth rate g, Δ_d, for decay rate d_{av}, and Δ_V for peak shedding $V_{\max}$. We did not treat effects as variant-specific, but rather sought to estimate the average sex differences in infection dynamics across the two variants tested.

2.8 Individual heterogeneity in disease course

To account for the fact that individuals have heterogeneous disease courses, we made our model hierarchical, with core virological parameter values for specific individuals distributed about the typical population values. If infected with a variant i, animal j has individual values for the virus growth rate g_{ij}, the virus decay rate d_{avij}, the peak load time t_a, and the peak load $V_{\max_{ij}}$.

These values are log-normally distributed about the population values for the given variant and animal sex, with estimated variant-specific standard deviations σ_{gi}, σ_{di}, σ_{ti} and σ_{Vi}. We use s_j as an indicator for the sex of hamster j (0 if female, 1 if male). Then:

$$\begin{aligned}
\log[g_{ij}] &\sim \text{Normal}(\log[g_i] + s_j\Delta_g, \sigma_{gi}) \\
\log[d_{avij}] &\sim \text{Normal}(\log[d_{avi}] + s_j\Delta_d, \sigma_{di}) \\
\log[t_{aij}] &\sim \text{Normal}(\log[t_{ai}], \sigma_{ti}) \\
\log[V_{\max_{ij}}] &\sim \text{Normal}(\log[V_{\max_i}] + s_j\Delta_V, \sigma_{Vi})
\end{aligned} \tag{10}$$

We also allowed for individual heterogeneity in respiration rates: animal j has an individual time-averaged respiration rate m_j log-normally distributed about the population value m with an estimated standard deviation σ_m:

$$\log[m_j] \sim \text{Normal}(\log[m] + s_j \Delta_m, \sigma_m) \tag{11}$$

3 Predicting observable quantities

We measured the following quantities:

- Virus subgenomic RNA (sgRNA) in oral swabs, measured via quantitative PCR (qPCR) in units of estimated copy numbers.
- Infectious virus in oral swabs, measured via endpoint titration in units of $\log_{10}$ TCID$_{50}$ per mL.
- Respiration rates, measured via plethysmography in units of mL air exhaled per unit time.
- Virus sgRNA collected on cage air filters over 24h sampling periods, measured via qPCR in units of estimated copy numbers.
- Infectious virus collected on cage air filters over 24h sampling periods, measured via plaque assay as total plaques formed.
- Infection statuses for each variant for each sentinel hamster.

3.1 Units of virus dynamics

We expressed $V_a(t)$ and $V_o(t)$ in units of total filter-collectible plaque forming units (PFU) shed per mL h^{-1} (i.e. units that directly predicting the cage air infectious virus measurements).

As discussed in section 2, our model explicitly relates infectious virus dynamics $V_a(t)$ and $V_o(t)$ to sgRNA copy number dynamics $N_a(t)$ and $N_o(t)$. The distinct conversion factors a_n and o_n and decay rates d_{an} and d_{on} for airborne versus oral shedding account for two types of possible differences between airborne and oral samples: biological differences (distinct underlying relationships between infectious virus concentration and sgRNA concentration) and measurement differences (distinct quantities of absolute sgRNA quantity recovered given the same underlying sgRNA concentration).

3.2 Converting predicted swab virus to units of TCID

To fit our model, we needed to convert our internal representation of predicted oral shedding of virus $V_o(t)$, which has the same "predicted air plaques" units as airborne shed virus $V_a(t)$, into the units in which we measured oral shedding: infectious virus $v_o(t)$ in units of $\log_{10}$ TCID$_{50}$/mL). We modeled this conversion via a multiplier o_v:

$$v_o(t) = o_v V_o(t) \tag{12}$$

The multiplier o_v subsumes both unit conversion and any actual multiplicative difference in virion numbers between airborne shedding and swabs (which could come from lower peak virion concentrations, sampling volume, et cetera.).

3.3 Predicting air sample plaques

We measured shedding into the air at the cage level; multiple hamsters were housed within a single cage. Moreover, samples were cumulative 24h accumulation on the air filter, rather than a point-sample.

So to fit our model, we needed to compute the cumulative airborne shedding A over some time period (t_0, t_1):

$$A(t_0, t_1) = \int_{t_0}^{t_1} m(t) V_a(t) \, \mathrm{d}t \tag{13}$$

where $m(t)$ is the animal's respiration rate. Integrating yields:

$$A(t_0, t_1) = \begin{cases} \frac{m}{g} \left(V_a(t_1) - V_a(t_0) \right) & t_1 \leq t_a \\ \\ \frac{m}{d_{av}} \left(V_a(t_0) - V_a(t_1) \right) & t_0 > t_a \\ \\ \frac{m}{g} \Big(V_a(t_a) - V_a(t_0) \Big) + \frac{m}{d_{av}} \left(V_a(t_a) - V_a(t_1) \right) & \text{o.w.} \end{cases} \tag{14}$$

where m is an appropriately-chosen constant to represent the time-varying effect of $m(t)$ on the value of the integral. Note that while ideally we would know how $m(t)$ and $V_a(t)$ change together and compute the integral explicitly, in practice we could only measure $m(t)$ coarsely, and so it was simpler to infer the appropriate value, understanding that it would not necessarily equal a naive temporal average.

Furthermore, since the air shedding measured accumulation on the air filter over a 24 hour period, we had to account for decay of infectious virus between exhalation and quantification. To do this, we assumed that the virus decays exponential in aerosols (as we have previously measured empirically[1]) but that minimal virus is lost once the filter is removed for virus quantification.

Suppose the the sampling period begins at a time t_0 post-infection and ends at a time t_1 when the filter is removed. Each hamster j sheds infectious virus at a rate $m V_{aj}(t)$ per unit time. But if the virus loses infectivity according to an exponential decay process with rate λ, then only a fraction $e^{-\lambda(t_s - t)}$ of the virions shed at time $t > t_0$ and collected on the filter will remain infectious when the filter is collected at t_1.

So the cumulative number of virions shed from t_0 to t_1 that remain infectious at t_1 is given by:

$$A_v(t_0, t_1) = m \int_{t_0}^{t_1} V_a(t) e^{-\lambda(t_1 - t)}\, \mathrm{d}t \tag{15}$$

This can be computed using the following antiderivative:

$$F(t, a, b) = \int \exp[at - \lambda(b - t)]\, \mathrm{d}t = \frac{1}{a + \lambda} \exp[at - \lambda(b - t)] \tag{16}$$

For convenience, define $\Delta t = t_1 - t_0$. If $t_1 < t_a$ (entire sample happens before peak airborne shedding), then:

$$A_v(t_0, t_1) = m V_a(t_0) \left[F(\Delta t, g, \Delta t) - F(0, g, \Delta t)\right] \tag{17}$$

Similarly, if $t_0 > t_a$ (entire sample taken after the peak), then:

$$A_v(t_0, t_1) = m V_a(t_0) \left[F(\Delta t, -d_{av}, \Delta t) - F(0, -d_{av}, \Delta t)\right] \tag{18}$$

If the peak occurs during sampling ($t_0 \leq t_a \leq t_1$), the problem can be solved piecewise:

$$\begin{aligned} A_v(t_0, t_1) =& m V_a(t_0) \left[F(t_a - t_0, g, \Delta t) - F(0, g, \Delta t)\right] + \\ & m V_a(t_a) \left[F(t_1 - t_a, -d_{av}, t_1 - t_a) - F(0, -d_{av}, t_1 - t_a)\right] \end{aligned} \tag{19}$$

3.4 Predicting air sample sgRNA

For air sample sgRNA, we again predicted cumulative accumulation. We did not model environmental degradation of detectable sgRNA, but rather chose to treat it implicitly via the decay rate ratio q_n relating airborne infectious virus shedding to airborne sgRNA shedding. We chose not to model sgRNA environmental degregation more explicitly because environmental half-lives for sgRNA are less well-characterized, but likely longer, than environmental half-lives for infectious virus.

The cumulative predicted number of sgRNA copies collected is:

$$A_n(t_0, t_1) = m \int_{t_0}^{t_1} N_a(t)\, \mathrm{d}t \tag{20}$$

A_n can be computed using the following antiderivative:

$$G(t, a) = \int \exp(at)\, \mathrm{d}t = \frac{1}{a} \exp(at) \tag{21}$$

Note that $G(t, a) = F(t, a, b)$ when $\lambda = 0$ (i.e. no environmental decay) or $b(t) = t$ (i.e. continuous and instantaneous collection leaving no time for environmental decay).

Again define $\Delta t = t_1 - t_0$. Then, similarly to 16:

$$A_n(t_0, t_1) = \begin{cases} mN_a(t_0)\left[G(\Delta t, g) - G(0, g)\right] & t_1 < t_a \\ \\ mN_a(t_0)\left[G(\Delta t, -d) - G(0, -d)\right] & t_0 > t_a \\ \\ mN_a(t_0)\left[G(t_a - t_0, g) - G(0, g)\right] + & t_0 \leq t_a \leq t_1 \\ mN_a(t_a)\left[G(t_1 - t_a, -d) - G(0, -d)\right] \end{cases} \tag{22}$$

3.5 Predicting sentinel exposures

Using our kinetics model, we were able to estimate probability each donor in our dual donor experiment had of infecting each sentinel, taking into account donor sex, infecting variant, and timing of exposure. This also enabled us to assess whether the absence of observed co-infections in sequential donor experiments was more suggestive of competitive interference or non-interference among the virus variants (see section 6 for methods and results).

To do this, we assumed that each sentinel's dose from each donor was proportional to the cumulative airborne shedding by the donor over the period of sentinel exposure. Given the short exposure period, we ignored the effect of environmental loss, so the computation was $A_v(t_0, t_1)$ as in equation 15, but with the environmental loss rate set to $\lambda = 0$. We assumed that the total dose received by each sentinel was equal to the cumulative shedding multiplied by an estimated variant-specific constant c_i that subsumes uncertainty about, sentinel respiration rate, cage airflow, and per-virion infectivity when inhaled by a hamster (since A_v has units of predicted *cell culture* plaques, and hamster airways may be more or less susceptible.

So in our model, each sentinel j receives a dose h_{ij} of variant i that depends on the virus shedding $A_{vij}(t_{0i}, t_{1i})$ from the donor animal associated to variant i and sentinel j, where t_{0i} and t_{1i} are the start and end times of the exposure:

$$h_{ij} = c_i A_{vij}(t_{0i}, t_{1i}) \tag{23}$$

We again applied a Poisson single-hit model of infection, so the probability $p_{\text{inf}}(i, j)$ that sentinel j is infected with variant i depends on the cumulative dose h_{ij} as:

$$p_{\text{inf}}(i, j) = 1 - e^{-h_{ij}} \tag{24}$$

4 Relating predicted quantities to observed quantities

4.1 Oral swabs

4.1.1 Infectious virus titers

Denote the kth measured oral swab titer by y_{vok}. Suppose it was sampled from individual animal j at time t. Then its predicted value is $v_{ok} = o_v V_{oj}(t)$.

We modeled the distribution of observed swab titers given $\log_{10}$TCID$_{50}$ values v_{ok} via a Poisson single-hit process, as we have described previously[2,3] (in particular, see section 2.2 of SI of[2]). Briefly, in a Poisson single-hit model of virus titration, a Poisson-distributed number of virions successfully infect cells in each. The mean μ of this Poisson depends on the underlying sample virus concentration v (in $\log_{10}$TCID$_{50}$) and degree of dilution D (in $\log_{10}$ fold dilutions):

$$\mu = \log(2)10^{v-D} \tag{25}$$

The factor of $\log(2)$ converts from units of TCID$_{50}$ to units of successful virions.

A well will be positive for infection if at least one virion infects a cell, which occurs with probability:

$$1 - \exp\left(-\log(2)10^{v-D}\right) \tag{26}$$

A complication to the typical single-hit model in this case is that we only had total counts of positive wells rather than exact well identities, dilutions, and positive/negative status. To handle this, we used an approximate method that integrates the likelihood function over the most probable configurations of positive and negative wells that could generate an observed total count. We describe this method in section 8.1.

4.1.2 Subgenomic RNA

If y_{nok} is the kth measurement of oral swab sgRNA, sampled from animal j at time t, its predicted value is $n_{ok} = N_o(t)$. To account for different sampling procedures and qPCR runs for the donor animals used the dual donor experiments compared to the animals used in the kinetics experiments, for the donor animals we added an estimated offset term f to the log copy number: $\log[n_{ok}] = \log[N_o(t)] + f$

We modeled the observed $\log_{10}$ oral swab sgRNA copy numbers y_{nok} as distributed about their predicted values n_{ok}, with an estimated variant-specific standard deviation σ_{noi} (where i is the variant infecting animal j) and censoring at the minimum and maximum observable values (which are given by the particular sgRNA standard curve):

$$\log_{10}(y_{noj}) \sim \text{Censored Normal}(\log_{10}(n_{oj}), \sigma_{noi}, n_{\min}, n_{\max}) \tag{27}$$

4.2 Air samples

4.2.1 Plaques

We used equations 17, 18, and 19 to predict the number of plaques v_{ak} observed on each filter.

Note that this implies $V_a(t)$ has units of filter plaques produced per mL exhaled air per unit time (in the absence of environmental decay).

If an observed plaque count y_{avk} comes from an air sample taken between time t_0 and time t_1 from a cage with n_h hamsters infected with variant j, the corresponding predicted plaque count v_{ak} is:

$$v_{ak} = \sum_{u=1}^{n_h} A_{vu}(t_0, t_1) \tag{28}$$

where $A_{vu}(t_0, t_1)$ is $A_v(t_0, t_1)$ for the uth hamster.

Since *in vitro* cell infection is well-described by a Poisson single-hit process[4] (possibly with binomial thinning), we modeled the observed plaque counts y_{avk} as Poisson-distributed about their predicted values v_{ak}:

$$y_{vak} \sim \text{Poisson}(v_{ak}) \tag{29}$$

4.2.2 Subgenomic RNA

Similarly, we used equation 20 to predict the number of sgRNA copies n_{ak} that would be observed when sampling cage k from t_0 until t_1:

$$n_{ak} = \sum_{u=1}^{n_h} A_{nu}(t_0, t_1) \tag{30}$$

where A_{nu} is the cumulative sgRNA shedding function A_n for hamster u.

We model the observed $\log_{10}$ air sample sgRNA copy numbers $\log_{10}(y_{nak})$ as normally distributed about their predicted values $\log_{10}(n_{ak})$ with an estimated variant-specific standard deviation σ_{nai} and censoring at the minimum and maximum possible $\log_{10}$ estimated copy numbers (which depend on the standard curve):

$$\log_{10}(y_{nak}) \sim \text{Censored Normal}(\log_{10}[n_{ak}], \sigma_{nai}, n_{\min}, n_{\max}) \tag{31}$$

4.3 Respiration rates

We modeled the observed log respiration rates for animal j $\log(y_{mij})$ as distributed about the animal's typical log value $\log(m_j)$ with a estimated standard deviation σ_r:

$$\log(y_{mij}) \sim \text{Normal}(\log(m_j), \sigma_r) \tag{32}$$

4.4 Sentinel infection status

Our dynamical model generates predicted infection probabilities $p_{\text{inf}}(i, j)$ for sentinel i with variant j (see section 3.5).

The observed infection status for sentinel j with variant i, $y_{pij} \in \{0, 1\}$ is therefore Bernoulli distributed with probability $p_{\text{inf}}[i, j]$:

$$y_{pij} \sim \text{Bernoulli}(p_{\text{inf}}[i, j]) \tag{33}$$

5 Prior distributions

In general, we sought to set prior distributions for our parameters that were "weakly informative"[5]; that is, that rule out biologically implausible or impossible values while remaining fairly agnostic about possible values of interest. We assessed the robustness of our prior distribution choices via prior predictive checks.

5.1 Respiration rates

We placed a normal prior on the population-wide mean log respiration rate $\log(m)$, with m in units of mL h^{-1}:

$$\log(m) \sim \text{Normal}(\log[4800], 0.25) \tag{34}$$

We place a positive-constrained normal prior on the individual respiration rate standard deviation σ_{mi} (see equation 10).

$$\sigma_{mi} \sim \text{PosNormal}(0, 0.25) \tag{35}$$

5.2 Virological parameters

We placed log-normal priors on the variant-specific time to peak t_{ai} and peak shedding rate $V_{\max_i}$; i indexes the variant. To encode prior information about the variant-specific growth and decay rates g_i and d_{avi} in an interpretable manner, we placed normal priors on the doubling and halving times (in hours) $t_{2i} = \log(2)/g_i$ and $t_{\frac{1}{2}i} = \log(2)/d_{avi}$ and then back-calculated g_i and d_{avi}.

$$\begin{aligned}
\log[t_{ai}] &\sim \text{Normal}(\log[24], 0.5) \\
\log[V_{\max_i}] &\sim \text{Normal}(\log[1] - \log[24] - \log[4800], 3) \\
\log[t_{2i}] &\sim \text{Normal}(\log[5], 0.5) \\
\log[t_{\frac{1}{2}i}] &\sim \text{Normal}(\log[15], 0.75) \\
\log[t_{\frac{1}{2}i}] &\sim \text{Normal}(\log[15], 0.75)
\end{aligned} \tag{36}$$

The prior mean for $\log[V_{\max_i}]$ can be interpreted as corresponding to the amount of shedding that would lead to 1 plaque(s) on the air filter from a 24h sample at the prior mean respiration rate of $\log[4800\text{mL h}^{-1}]$.

We placed normal priors on the male sex effects Δ_m, Δ_g, Δ_d, and Δ_V that modify the virological parameters:

$$\begin{aligned}
\Delta_m &\sim \text{Normal}(0, 0.25) \\
\Delta_g &\sim \text{Normal}(0, 0.25) \\
\Delta_d &\sim \text{Normal}(0, 0.25) \\
\Delta_V &\sim \text{Normal}(0, 0.25)
\end{aligned} \tag{37}$$

We placed lognormal priors priors on the swab to air peak timing ratio ω, the swab to air decay rate ratio q_o, the sgRNA to infectious virus decay rate ratio q_n, the air infectious virus to oral TCID conversion factor o_v, the air and swab copy number to infectious virus ratios

a_n and o_n. We placed a normal prior on the donor log copy number offset f:

$$\begin{aligned} \log(\omega) &\sim \text{Normal}(0, 0.25) \\ \log(q_o) &\sim \text{Normal}(0, 1) \\ \log(q_n) &\sim \text{Normal}(\log[1], 1) \\ \log(o_v) &\sim \text{Normal}(0, 10) \\ \log(a_n) &\sim \text{Normal}(0, 10) \\ \log(o_n) &\sim \text{Normal}(0, 10) \\ f &\sim \text{Normal}(0, 1.5) \end{aligned} \tag{38}$$

We placed positive-constrained normal priors on the hierarchical standard deviations that specify degree of individual variation about these population-wide virological parameters:

$$\begin{aligned} \sigma_{gi} &\sim \text{Normal}(0, 0.2) \\ \sigma_{di} &\sim \text{Normal}(0, 0.2) \\ \sigma_{ti} &\sim \text{Normal}(0, 0.15) \\ \sigma_{Vi} &\sim \text{Normal}(0, 2) \end{aligned} \tag{39}$$

5.3 Sentinel infection process constant

We placed a lognormal prior on the variant-specific sentinel infection process constant c_i:

$$\log[c_i] \sim \text{Normal}(0, 3) \tag{40}$$

5.4 Observation error standard deviations

We placed positive-constrained normal priors on the observation process standard deviations for respiration rate σ_r (equation 32), oral swab sgRNA copies σ_{noi} (equation 27), air sample sgRNA copies σ_{nai}:

$$\begin{aligned} \sigma_r &\sim \text{PosNormal}(0, 0.2) \\ \sigma_{noi} &\sim \text{PosNormal}(0, 0.5) \\ \sigma_{nai} &\sim \text{PosNormal}(0, 0.5) \end{aligned} \tag{41}$$

6 Assessing coinfection probabilities

To assess the probability of coinfection, we visualized the infection probabilities for each variant in each cage. Given no interaction between two variants' infection processes, the probability of being coinfected for each hamster j is the product of the hamster's probabilities for each variant:

$$P_{\text{coinf}}(j) = P_{\text{inf}}(1, j) P_{\text{inf}}(2, j) \tag{42}$$

The distribution of the number of coinfections in a given cage or experiment is then the convolution of these individual Bernoulli-distributed outcomes for individuals.

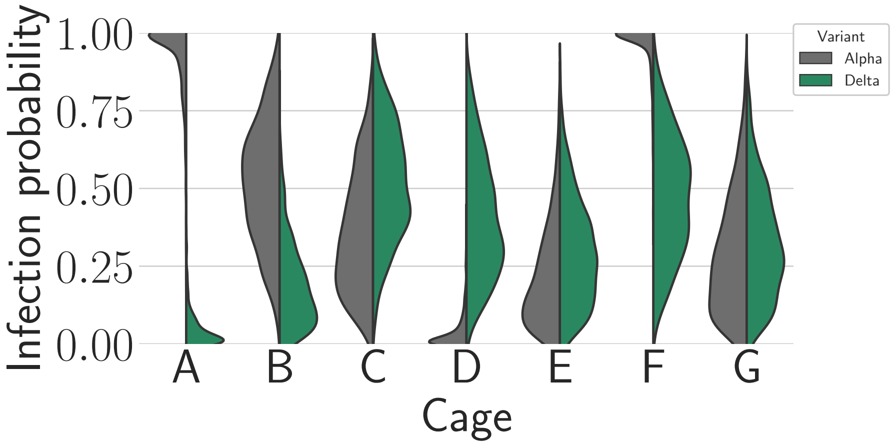

Figure S1: Posterior estimates for the infection probabilities for each variant in each cage. Half-violin plots show posterior densities for Alpha infection probability (gray) compared to Delta infection probability (green). There were very high Alpha infection probabilities in cages A and F.

6.1 Results

Figure S1 shows the estimated infection probabilities by variant and cage. Cage F was the only cage in which we observed coinfections, and our model shows that it is indeed the only cage in which both Alpha and Delta clearly had a high probability of causing infections in the sentinels.

We then calculated the posterior estimated probability of coinfection occuring for each hamster in each cage, according to equation 42. Figure S2 shows the resulting estimates.

The model estimates that coinfection probabilities were highest in Cage F simply because both Alpha and Delta infection probabilities were high. In other cages, coinfection probabilities are substantially lower, since at least one variant has a low individual infection probability S1. Cage C (Delta, then Alpha) is the only sequential exposure cage in which the absence of coinfections is perhaps surprising; even there, the data are consistent with a coinfection probability of under 25% or even under 10%, so given that only 5 sentinels were exposed, the absence of a coinfection is consistent with random variation.

Finally, to assess whether the absence of any coinfections in the sequential experiments while several were observed in the simultaneous experiments could be explained by chance, we calculated the posterior distribution for the expected number of coinfections by experiment type (this is the sum of the probability for each cage times the number of sentinels in that cage). The results are shown in Figure S3

The model suggests that the absence of coinfections in the sequential exposures could simply result from low probabilities in all cages except C; the data are consistent with an expectation of between zero and two coinfections, though more than that would also have been plausible.

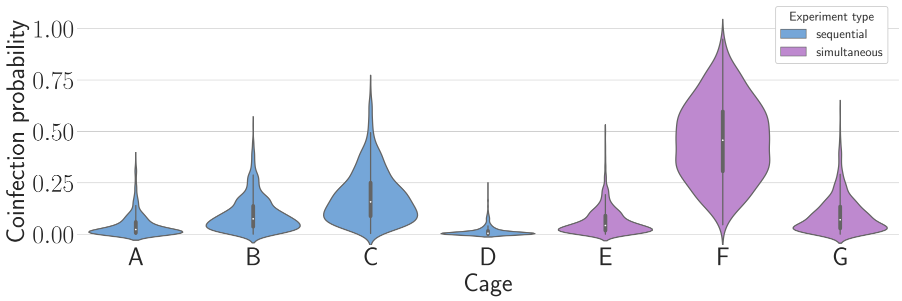

Figure S2: Posterior estimates for coinfection probabilities by cage. Sequential exposures shown in blue, simultaneous exposures shown in pink. Cage F, where coinfections were actually observed, has a substantially higher coinfection probability estimate than other cages.

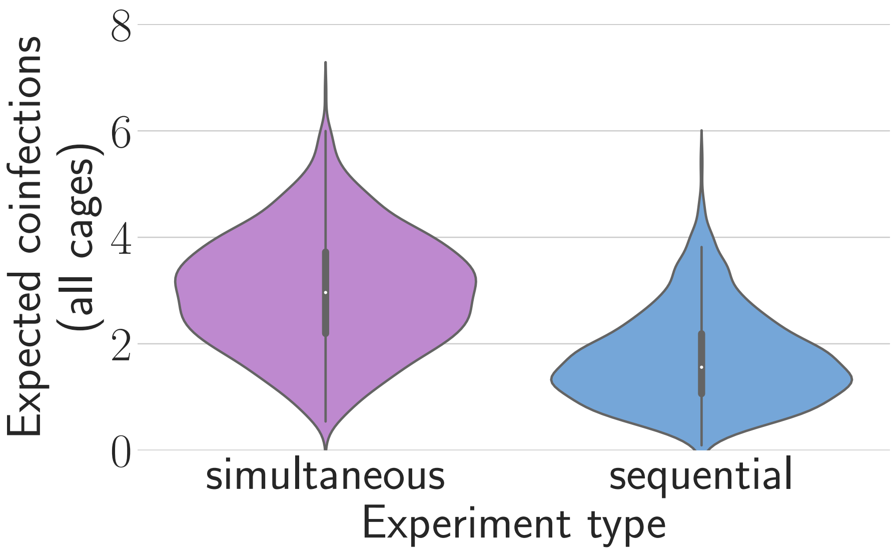

Figure S3: Expected coinfection counts for sequential and simultaneous experiments.

Taken together, our results suggest that differences in donor virus dynamics and shedding could readily explain the differences between sequential and simultaneous exposures in our small-N dataset. Identifying or ruling out competitive (or facilitating) interaction among virus variants during sequential versus simultaneous transmission would likely require larger samples.

7 Computational methods

We implemented and conducted inference from our model in Python using the Numpyro probabilistic programming framework[6]. We drew posterior samples using Numpyro's iterative implementation[6] of the No-U-Turn Sampler (NUTS)[7], a form of Hamiltonian Monte Carlo (HMC).

For inference purposes, we set a minimum rate of 10^{-20} for all Poisson distributions of "hitting" virions (for filter plaques and virus titration. True 0 rates can cause numerical issue when conducting NUTS sampling with Numpyro. This minimum rate of 10^{-20} can be thought of as representing a very small probability of a false positive plaque or well.

We prepared data for modeling and analyzed and visualized output in Python[8]; the packages Numpy[9], Scipy[10], Matplotlib[11], and Polars[12] were particularly critical.

All code and data to reproduce Bayesian inference results, including model fits and model output figures, is available on the project Github repository (https://example.com) and archived on Zenodo (https://example.com).

8 Additional mathematical details

8.1 Well observation process

But since Spearman-Karber estimates for a 0.1 mL inoculum give an exact value for the total number of positive wells in a 4 well by 8 dilution series, we were able to back calculate the total number of positive wells. By further assuming that all dilutions with any positive wells were all positive except for the last two rows (these are by far the most probable way to produce a given number, we were then able to calculate the approximate likelihood of observing a given number of positive wells n given a true underlying titer v. We first calculate the implied dilution for the penultimate row, d, and the implied number k of total positives at dilutions d and $d + 1$:

$$\begin{aligned} k &= \min\{n, n \ 4 + \text{mod } 4\} \\ d &= \frac{n-k}{4} \end{aligned} \tag{43}$$

where mod denotes the modulo operation (remainder when n is divided by 4).

Then the approximate log likelihood for observing n positive wells given an underlying virus concentration v is the sum over the possible ways to generate k positives at dilutions d and $d + 1$. Define the random variables K_d and K_{d+1} as the number of wells positive at dilutions d and $d + 1$, respectively. The the probability of observing a certain value of K_d given the underlying virus concentration v is given by a binomial distribution with success probability equal to the single hit probability at dilution d, i.e.:

$$P(K_d = k \mid v) = \binom{4}{k} p^k (1-p)^{4-k} \tag{44}$$

with $p = 1 - \exp\left(-\log(2)10^{v-d}\right)$. And so:

$$\mathcal{L}(n \mid v) \approx \sum_{c=k-4}^{4} \log[P(K_d = c \mid v)] + \log[P(K_{d+1} = k - c \mid v)] \tag{45}$$

References

1. Van Doremalen, N. *et al.* Aerosol and surface stability of SARS-CoV-2 as compared with SARS-CoV-1. *New England Journal of Medicine* **382,** 1564–1567 (2020).
2. Gamble, A. *et al.* Heat-treated virus inactivation rate depends strongly on treatment procedure: illustration with SARS-CoV-2. *Applied and environmental microbiology* **87,** e00314–21 (2021).
3. Morris, D. H. *et al.* Mechanistic theory predicts the effects of temperature and humidity on inactivation of SARS-CoV-2 and other enveloped viruses. *Elife* **10,** e65902 (2021).
4. Brownie, C. *et al.* Estimating viral titres in solutions with low viral loads. *Biologicals* **39,** 224–230 (2011).
5. Gelman, A., Jakulin, A., Pittau, M. G. & Su, Y.-S. A weakly informative default prior distribution for logistic and other regression models. *The annals of applied statistics* **2,** 1360–1383 (2008).
6. Phan, D., Pradhan, N. & Jankowiak, M. Composable effects for flexible and accelerated probabilistic programming in NumPyro. *arXiv preprint arXiv:1912.11554* (2019).
7. Hoffman, M. D., Gelman, A., *et al.* The No-U-Turn sampler: adaptively setting path lengths in Hamiltonian Monte Carlo. *J. Mach. Learn. Res.* **15,** 1593–1623 (2014).
8. Van Rossum, G. & Drake, F. L. *Python 3 Reference Manual* ISBN: 1441412697 (CreateSpace, Scotts Valley, CA, 2009).
9. Harris, C. R. *et al.* Array programming with NumPy. *Nature* **585,** 357–362. https://doi.org/10.1038/s41586-020-2649-2 (Sept. 2020).
10. Jones, E., Oliphant, T., Peterson, P., *et al. SciPy: Open source scientific tools for Python* 2001. http://www.scipy.org/.
11. Hunter, J. D. Matplotlib: A 2D graphics environment. *Computing in Science & Engineering* **9,** 90–95 (2007).
12. Vink, R. *Polars: Blazingly fast DataFrames in Rust, Python & Node.js* https://github.com/pola-rs/polars.
